# Measurement of the inclusive 3-jet production differential cross section in proton–proton collisions at 7 TeV and determination of the strong coupling constant in the TeV range

**DOI:** 10.1140/epjc/s10052-015-3376-y

**Published:** 2015-05-01

**Authors:** V. Khachatryan, A. M. Sirunyan, A. Tumasyan, W. Adam, T. Bergauer, M. Dragicevic, J. Erö, C. Fabjan, M. Friedl, R. Frühwirth, V. M. Ghete, C. Hartl, N. Hörmann, J. Hrubec, M. Jeitler, W. Kiesenhofer, V. Knünz, M. Krammer, I. Krätschmer, D. Liko, I. Mikulec, D. Rabady, B. Rahbaran, H. Rohringer, R. Schöfbeck, J. Strauss, A. Taurok, W. Treberer-Treberspurg, W. Waltenberger, C.-E. Wulz, V. Mossolov, N. Shumeiko, J. Suarez Gonzalez, S. Alderweireldt, M. Bansal, S. Bansal, T. Cornelis, E. A. De Wolf, X. Janssen, A. Knutsson, S. Luyckx, S. Ochesanu, R. Rougny, M. Van De Klundert, H. Van Haevermaet, P. Van Mechelen, N. Van Remortel, A. Van Spilbeeck, F. Blekman, S. Blyweert, J. D’Hondt, N. Daci, N. Heracleous, J. Keaveney, S. Lowette, M. Maes, A. Olbrechts, Q. Python, D. Strom, S. Tavernier, W. Van Doninck, P. Van Mulders, G. P. Van Onsem, I. Villella, C. Caillol, B. Clerbaux, G. De Lentdecker, D. Dobur, L. Favart, A. P. R. Gay, A. Grebenyuk, A. Léonard, A. Mohammadi, L. Perniè, T. Reis, T. Seva, L. Thomas, C. Vander Velde, P. Vanlaer, J. Wang, F. Zenoni, V. Adler, K. Beernaert, L. Benucci, A. Cimmino, S. Costantini, S. Crucy, S. Dildick, A. Fagot, G. Garcia, J. Mccartin, A. A. Ocampo Rios, D. Ryckbosch, S. Salva Diblen, M. Sigamani, N. Strobbe, F. Thyssen, M. Tytgat, E. Yazgan, N. Zaganidis, S. Basegmez, C. Beluffi, G. Bruno, R. Castello, A. Caudron, L. Ceard, G. G. Da Silveira, C. Delaere, T. du Pree, D. Favart, L. Forthomme, A. Giammanco, J. Hollar, A. Jafari, P. Jez, M. Komm, V. Lemaitre, C. Nuttens, D. Pagano, L. Perrini, A. Pin, K. Piotrzkowski, A. Popov, L. Quertenmont, M. Selvaggi, M. Vidal Marono, J. M. Vizan Garcia, N. Beliy, T. Caebergs, E. Daubie, G. H. Hammad, W. L. Aldá Júnior, G. A. Alves, L. Brito, M. Correa Martins Junior, T. Dos Reis Martins, C. Mora Herrera, M. E. Pol, W. Carvalho, J. Chinellato, A. Custódio, E. M. Da Costa, D. De Jesus Damiao, C. De Oliveira Martins, S. Fonseca De Souza, H. Malbouisson, D. Matos Figueiredo, L. Mundim, H. Nogima, W. L. Prado Da Silva, J. Santaolalla, A. Santoro, A. Sznajder, E. J. Tonelli Manganote, A. Vilela Pereira, C. A. Bernardes, S. Dogra, T. R. Fernandez Perez Tomei, E. M. Gregores, P. G. Mercadante, S. F. Novaes, Sandra S. Padula, A. Aleksandrov, V. Genchev, P. Iaydjiev, A. Marinov, S. Piperov, M. Rodozov, S. Stoykova, G. Sultanov, V. Tcholakov, M. Vutova, A. Dimitrov, I. Glushkov, R. Hadjiiska, V. Kozhuharov, L. Litov, B. Pavlov, P. Petkov, J. G. Bian, G. M. Chen, H. S. Chen, M. Chen, R. Du, C. H. Jiang, R. Plestina, F. Romeo, J. Tao, Z. Wang, C. Asawatangtrakuldee, Y. Ban, Q. Li, S. Liu, Y. Mao, S. J. Qian, D. Wang, W. Zou, C. Avila, L. F. Chaparro Sierra, C. Florez, J. P. Gomez, B. Gomez Moreno, J. C. Sanabria, N. Godinovic, D. Lelas, D. Polic, I. Puljak, Z. Antunovic, M. Kovac, V. Brigljevic, K. Kadija, J. Luetic, D. Mekterovic, L. Sudic, A. Attikis, G. Mavromanolakis, J. Mousa, C. Nicolaou, F. Ptochos, P. A. Razis, M. Bodlak, M. Finger, M. Finger, Y. Assran, A. Ellithi Kamel, M. A. Mahmoud, A. Radi, M. Kadastik, M. Murumaa, M. Raidal, A. Tiko, P. Eerola, G. Fedi, M. Voutilainen, J. Härkönen, V. Karimäki, R. Kinnunen, M. J. Kortelainen, T. Lampén, K. Lassila-Perini, S. Lehti, T. Lindén, P. Luukka, T. Mäenpää, T. Peltola, E. Tuominen, J. Tuominiemi, E. Tuovinen, L. Wendland, J. Talvitie, T. Tuuva, M. Besancon, F. Couderc, M. Dejardin, D. Denegri, B. Fabbro, J. L. Faure, C. Favaro, F. Ferri, S. Ganjour, A. Givernaud, P. Gras, G. Hamel de Monchenault, P. Jarry, E. Locci, J. Malcles, J. Rander, A. Rosowsky, M. Titov, S. Baffioni, F. Beaudette, P. Busson, C. Charlot, T. Dahms, M. Dalchenko, L. Dobrzynski, N. Filipovic, A. Florent, R. Granier de Cassagnac, L. Mastrolorenzo, P. Miné, C. Mironov, I. N. Naranjo, M. Nguyen, C. Ochando, P. Paganini, S. Regnard, R. Salerno, J. B. Sauvan, Y. Sirois, C. Veelken, Y. Yilmaz, A. Zabi, J.-L. Agram, J. Andrea, A. Aubin, D. Bloch, J.-M. Brom, E. C. Chabert, C. Collard, E. Conte, J.-C. Fontaine, D. Gelé, U. Goerlach, C. Goetzmann, A.-C. Le Bihan, P. Van Hove, S. Gadrat, S. Beauceron, N. Beaupere, G. Boudoul, E. Bouvier, S. Brochet, C. A. Carrillo Montoya, J. Chasserat, R. Chierici, D. Contardo, P. Depasse, H. El Mamouni, J. Fan, J. Fay, S. Gascon, M. Gouzevitch, B. Ille, T. Kurca, M. Lethuillier, L. Mirabito, S. Perries, J. D. Ruiz Alvarez, D. Sabes, L. Sgandurra, V. Sordini, M. Vander Donckt, P. Verdier, S. Viret, H. Xiao, I. Bagaturia, C. Autermann, S. Beranek, M. Bontenackels, M. Edelhoff, L. Feld, O. Hindrichs, K. Klein, A. Ostapchuk, A. Perieanu, F. Raupach, J. Sammet, S. Schael, H. Weber, B. Wittmer, V. Zhukov, M. Ata, M. Brodski, E. Dietz-Laursonn, D. Duchardt, M. Erdmann, R. Fischer, A. Güth, T. Hebbeker, C. Heidemann, K. Hoepfner, D. Klingebiel, S. Knutzen, P. Kreuzer, M. Merschmeyer, A. Meyer, P. Millet, M. Olschewski, K. Padeken, P. Papacz, H. Reithler, S. A. Schmitz, L. Sonnenschein, D. Teyssier, S. Thüer, M. Weber, V. Cherepanov, Y. Erdogan, G. Flügge, H. Geenen, M. Geisler, W. Haj Ahmad, A. Heister, F. Hoehle, B. Kargoll, T. Kress, Y. Kuessel, A. Künsken, J. Lingemann, A. Nowack, I. M. Nugent, L. Perchalla, O. Pooth, A. Stahl, I. Asin, N. Bartosik, J. Behr, W. Behrenhoff, U. Behrens, A. J. Bell, M. Bergholz, A. Bethani, K. Borras, A. Burgmeier, A. Cakir, L. Calligaris, A. Campbell, S. Choudhury, F. Costanza, C. Diez Pardos, S. Dooling, T. Dorland, G. Eckerlin, D. Eckstein, T. Eichhorn, G. Flucke, J. Garay Garcia, A. Geiser, P. Gunnellini, J. Hauk, M. Hempel, D. Horton, H. Jung, A. Kalogeropoulos, M. Kasemann, P. Katsas, J. Kieseler, C. Kleinwort, D. Krücker, W. Lange, J. Leonard, K. Lipka, A. Lobanov, W. Lohmann, B. Lutz, R. Mankel, I. Marfin, I.-A. Melzer-Pellmann, A. B. Meyer, G. Mittag, J. Mnich, A. Mussgiller, S. Naumann-Emme, A. Nayak, O. Novgorodova, E. Ntomari, H. Perrey, D. Pitzl, R. Placakyte, A. Raspereza, P. M. Ribeiro Cipriano, B. Roland, E. Ron, M. Ö. Sahin, J. Salfeld-Nebgen, P. Saxena, R. Schmidt, T. Schoerner-Sadenius, M. Schröder, C. Seitz, S. Spannagel, A. D. R. Vargas Trevino, R. Walsh, C. Wissing, M. Aldaya Martin, V. Blobel, M. Centis Vignali, A. R. Draeger, J. Erfle, E. Garutti, K. Goebel, M. Görner, J. Haller, M. Hoffmann, R. S. Höing, H. Kirschenmann, R. Klanner, R. Kogler, J. Lange, T. Lapsien, T. Lenz, I. Marchesini, J. Ott, T. Peiffer, N. Pietsch, J. Poehlsen, T. Poehlsen, D. Rathjens, C. Sander, H. Schettler, P. Schleper, E. Schlieckau, A. Schmidt, M. Seidel, V. Sola, H. Stadie, G. Steinbrück, D. Troendle, E. Usai, L. Vanelderen, A. Vanhoefer, C. Barth, C. Baus, J. Berger, C. Böser, E. Butz, T. Chwalek, W. De Boer, A. Descroix, A. Dierlamm, M. Feindt, F. Frensch, M. Giffels, F. Hartmann, T. Hauth, U. Husemann, I. Katkov, A. Kornmayer, E. Kuznetsova, P. Lobelle Pardo, M. U. Mozer, Th. Müller, A. Nürnberg, G. Quast, K. Rabbertz, F. Ratnikov, S. Röcker, G. Sieber, H. J. Simonis, F. M. Stober, R. Ulrich, J. Wagner-Kuhr, S. Wayand, T. Weiler, R. Wolf, G. Anagnostou, G. Daskalakis, T. Geralis, V. A. Giakoumopoulou, A. Kyriakis, D. Loukas, A. Markou, C. Markou, A. Psallidas, I. Topsis-Giotis, A. Agapitos, S. Kesisoglou, A. Panagiotou, N. Saoulidou, E. Stiliaris, X. Aslanoglou, I. Evangelou, G. Flouris, C. Foudas, P. Kokkas, N. Manthos, I. Papadopoulos, E. Paradas, G. Bencze, C. Hajdu, P. Hidas, D. Horvath, F. Sikler, V. Veszpremi, G. Vesztergombi, A. J. Zsigmond, N. Beni, S. Czellar, J. Karancsi, J. Molnar, J. Palinkas, Z. Szillasi, P. Raics, Z. L. Trocsanyi, B. Ujvari, S. K. Swain, S. B. Beri, V. Bhatnagar, R. Gupta, U. Bhawandeep, A. K. Kalsi, M. Kaur, R. Kumar, M. Mittal, N. Nishu, J. B. Singh, Ashok Kumar, Arun Kumar, S. Ahuja, A. Bhardwaj, B. C. Choudhary, A. Kumar, S. Malhotra, M. Naimuddin, K. Ranjan, V. Sharma, S. Banerjee, S. Bhattacharya, K. Chatterjee, S. Dutta, B. Gomber, Sa. Jain, Sh. Jain, R. Khurana, A. Modak, S. Mukherjee, D. Roy, S. Sarkar, M. Sharan, A. Abdulsalam, D. Dutta, S. Kailas, V. Kumar, A. K. Mohanty, L. M. Pant, P. Shukla, A. Topkar, T. Aziz, S. Banerjee, S. Bhowmik, R. M. Chatterjee, R. K. Dewanjee, S. Dugad, S. Ganguly, S. Ghosh, M. Guchait, A. Gurtu, G. Kole, S. Kumar, M. Maity, G. Majumder, K. Mazumdar, G. B. Mohanty, B. Parida, K. Sudhakar, N. Wickramage, H. Bakhshiansohi, H. Behnamian, S. M. Etesami, A. Fahim, R. Goldouzian, M. Khakzad, M. Mohammadi Najafabadi, M. Naseri, S. Paktinat Mehdiabadi, F. Rezaei Hosseinabadi, B. Safarzadeh, M. Zeinali, M. Felcini, M. Grunewald, M. Abbrescia, L. Barbone, C. Calabria, S. S. Chhibra, A. Colaleo, D. Creanza, N. De Filippis, M. De Palma, L. Fiore, G. Iaselli, G. Maggi, M. Maggi, S. My, S. Nuzzo, A. Pompili, G. Pugliese, R. Radogna, G. Selvaggi, L. Silvestris, R. Venditti, G. Zito, G. Abbiendi, A. C. Benvenuti, D. Bonacorsi, S. Braibant-Giacomelli, L. Brigliadori, R. Campanini, P. Capiluppi, A. Castro, F. R. Cavallo, G. Codispoti, M. Cuffiani, G. M. Dallavalle, F. Fabbri, A. Fanfani, D. Fasanella, P. Giacomelli, C. Grandi, L. Guiducci, S. Marcellini, G. Masetti, A. Montanari, F. L. Navarria, A. Perrotta, A. M. Rossi, F. Primavera, T. Rovelli, G. P. Siroli, N. Tosi, R. Travaglini, S. Albergo, G. Cappello, M. Chiorboli, S. Costa, F. Giordano, R. Potenza, A. Tricomi, C. Tuve, G. Barbagli, V. Ciulli, C. Civinini, R. D’Alessandro, E. Focardi, E. Gallo, S. Gonzi, V. Gori, P. Lenzi, M. Meschini, S. Paoletti, G. Sguazzoni, A. Tropiano, L. Benussi, S. Bianco, F. Fabbri, D. Piccolo, R. Ferretti, F. Ferro, M. Lo Vetere, E. Robutti, S. Tosi, M. E. Dinardo, S. Fiorendi, S. Gennai, R. Gerosa, A. Ghezzi, P. Govoni, M. T. Lucchini, S. Malvezzi, R. A. Manzoni, A. Martelli, B. Marzocchi, D. Menasce, L. Moroni, M. Paganoni, D. Pedrini, S. Ragazzi, N. Redaelli, T. Tabarelli de Fatis, S. Buontempo, N. Cavallo, S. Di Guida, F. Fabozzi, A. O. M. Iorio, L. Lista, S. Meola, M. Merola, P. Paolucci, P. Azzi, N. Bacchetta, D. Bisello, A. Branca, R. Carlin, P. Checchia, M. Dall’Osso, T. Dorigo, M. Galanti, F. Gasparini, U. Gasparini, P. Giubilato, A. Gozzelino, K. Kanishchev, S. Lacaprara, M. Margoni, A. T. Meneguzzo, J. Pazzini, N. Pozzobon, P. Ronchese, F. Simonetto, E. Torassa, M. Tosi, S. Vanini, S. Ventura, P. Zotto, A. Zucchetta, M. Gabusi, S. P. Ratti, V. Re, C. Riccardi, P. Salvini, P. Vitulo, M. Biasini, G. M. Bilei, D. Ciangottini, L. Fanò, P. Lariccia, G. Mantovani, M. Menichelli, A. Saha, A. Santocchia, A. Spiezia, K. Androsov, P. Azzurri, G. Bagliesi, J. Bernardini, T. Boccali, G. Broccolo, R. Castaldi, M. A. Ciocci, R. Dell’Orso, S. Donato, G. Fedi, F. Fiori, L. Foà, A. Giassi, M. T. Grippo, F. Ligabue, T. Lomtadze, L. Martini, A. Messineo, C. S. Moon, F. Palla, A. Rizzi, A. Savoy-Navarro, A. T. Serban, P. Spagnolo, P. Squillacioti, R. Tenchini, G. Tonelli, A. Venturi, P. G. Verdini, C. Vernieri, L. Barone, F. Cavallari, G. D’imperio, D. Del Re, M. Diemoz, M. Grassi, C. Jorda, E. Longo, F. Margaroli, P. Meridiani, F. Micheli, S. Nourbakhsh, G. Organtini, R. Paramatti, S. Rahatlou, C. Rovelli, F. Santanastasio, L. Soffi, P. Traczyk, N. Amapane, R. Arcidiacono, S. Argiro, M. Arneodo, R. Bellan, C. Biino, N. Cartiglia, S. Casasso, M. Costa, A. Degano, N. Demaria, L. Finco, C. Mariotti, S. Maselli, E. Migliore, V. Monaco, M. Musich, M. M. Obertino, G. Ortona, L. Pacher, N. Pastrone, M. Pelliccioni, G. L. Pinna Angioni, A. Potenza, A. Romero, M. Ruspa, R. Sacchi, A. Solano, A. Staiano, U. Tamponi, S. Belforte, V. Candelise, M. Casarsa, F. Cossutti, G. Della Ricca, B. Gobbo, C. La Licata, M. Marone, A. Schizzi, T. Umer, A. Zanetti, S. Chang, T. A. Kropivnitskaya, S. K. Nam, D. H. Kim, G. N. Kim, M. S. Kim, M. S. Kim, D. J. Kong, S. Lee, Y. D. Oh, H. Park, A. Sakharov, D. C. Son, T. J. Kim, J. Y. Kim, S. Song, S. Choi, D. Gyun, B. Hong, M. Jo, H. Kim, Y. Kim, B. Lee, K. S. Lee, S. K. Park, Y. Roh, M. Choi, J. H. Kim, I. C. Park, G. Ryu, M. S. Ryu, Y. Choi, Y. K. Choi, J. Goh, D. Kim, E. Kwon, J. Lee, H. Seo, I. Yu, A. Juodagalvis, J. R. Komaragiri, M. A. B. Md Ali, H. Castilla-Valdez, E. De La Cruz-Burelo, I. Heredia-de La Cruz, A. Hernandez-Almada, R. Lopez-Fernandez, A. Sanchez-Hernandez, S. Carrillo Moreno, F. Vazquez Valencia, I. Pedraza, H. A. Salazar Ibarguen, E. Casimiro Linares, A. Morelos Pineda, D. Krofcheck, P. H. Butler, S. Reucroft, A. Ahmad, M. Ahmad, Q. Hassan, H. R. Hoorani, S. Khalid, W. A. Khan, T. Khurshid, M. A. Shah, M. Shoaib, H. Bialkowska, M. Bluj, B. Boimska, T. Frueboes, M. Górski, M. Kazana, K. Nawrocki, K. Romanowska-Rybinska, M. Szleper, P. Zalewski, G. Brona, K. Bunkowski, M. Cwiok, W. Dominik, K. Doroba, A. Kalinowski, M. Konecki, J. Krolikowski, M. Misiura, M. Olszewski, W. Wolszczak, P. Bargassa, C. Beir ao Da Cruz E Silva, P. Faccioli, P. G. Ferreira Parracho, M. Gallinaro, L. Lloret Iglesias, F. Nguyen, J. Rodrigues Antunes, J. Seixas, J. Varela, P. Vischia, S. Afanasiev, P. Bunin, M. Gavrilenko, I. Golutvin, I. Gorbunov, A. Kamenev, V. Karjavin, V. Konoplyanikov, A. Lanev, A. Malakhov, V. Matveev, P. Moisenz, V. Palichik, V. Perelygin, S. Shmatov, N. Skatchkov, V. Smirnov, A. Zarubin, V. Golovtsov, Y. Ivanov, V. Kim, P. Levchenko, V. Murzin, V. Oreshkin, I. Smirnov, V. Sulimov, L. Uvarov, S. Vavilov, A. Vorobyev, An. Vorobyev, Yu. Andreev, A. Dermenev, S. Gninenko, N. Golubev, M. Kirsanov, N. Krasnikov, A. Pashenkov, D. Tlisov, A. Toropin, V. Epshteyn, V. Gavrilov, N. Lychkovskaya, V. Popov, G. Safronov, S. Semenov, A. Spiridonov, V. Stolin, E. Vlasov, A. Zhokin, V. Andreev, M. Azarkin, I. Dremin, M. Kirakosyan, A. Leonidov, G. Mesyats, S. V. Rusakov, A. Vinogradov, A. Belyaev, E. Boos, M. Dubinin, L. Dudko, A. Ershov, A. Gribushin, V. Klyukhin, O. Kodolova, I. Lokhtin, S. Obraztsov, S. Petrushanko, V. Savrin, A. Snigirev, I. Azhgirey, I. Bayshev, S. Bitioukov, V. Kachanov, A. Kalinin, D. Konstantinov, V. Krychkine, V. Petrov, R. Ryutin, A. Sobol, L. Tourtchanovitch, S. Troshin, N. Tyurin, A. Uzunian, A. Volkov, P. Adzic, M. Ekmedzic, J. Milosevic, V. Rekovic, J. Alcaraz Maestre, C. Battilana, E. Calvo, M. Cerrada, M. Chamizo Llatas, N. Colino, B. De La Cruz, A. Delgado Peris, D. Domínguez Vázquez, A. Escalante Del Valle, C. Fernandez Bedoya, J. P. Fernández Ramos, J. Flix, M. C. Fouz, P. Garcia-Abia, O. Gonzalez Lopez, S. Goy Lopez, J. M. Hernandez, M. I. Josa, E. Navarro De Martino, A. Pérez-Calero Yzquierdo, J. Puerta Pelayo, A. Quintario Olmeda, I. Redondo, L. Romero, M. S. Soares, C. Albajar, J. F. de Trocóniz, M. Missiroli, D. Moran, H. Brun, J. Cuevas, J. Fernandez Menendez, S. Folgueras, I. Gonzalez Caballero, J. A. Brochero Cifuentes, I. J. Cabrillo, A. Calderon, J. Duarte Campderros, M. Fernandez, G. Gomez, A. Graziano, A. Lopez Virto, J. Marco, R. Marco, C. Martinez Rivero, F. Matorras, F. J. Munoz Sanchez, J. Piedra Gomez, T. Rodrigo, A. Y. Rodríguez-Marrero, A. Ruiz-Jimeno, L. Scodellaro, I. Vila, R. Vilar Cortabitarte, D. Abbaneo, E. Auffray, G. Auzinger, M. Bachtis, P. Baillon, A. H. Ball, D. Barney, A. Benaglia, J. Bendavid, L. Benhabib, J. F. Benitez, C. Bernet, G. Bianchi, P. Bloch, A. Bocci, A. Bonato, O. Bondu, C. Botta, H. Breuker, T. Camporesi, G. Cerminara, S. Colafranceschi, M. D’Alfonso, D. d’Enterria, A. Dabrowski, A. David, F. De Guio, A. De Roeck, S. De Visscher, E. Di Marco, M. Dobson, M. Dordevic, B. Dorney, N. Dupont-Sagorin, A. Elliott-Peisert, J. Eugster, G. Franzoni, W. Funk, D. Gigi, K. Gill, D. Giordano, M. Girone, F. Glege, R. Guida, S. Gundacker, M. Guthoff, J. Hammer, M. Hansen, P. Harris, J. Hegeman, V. Innocente, P. Janot, K. Kousouris, K. Krajczar, P. Lecoq, C. Lourenço, N. Magini, L. Malgeri, M. Mannelli, J. Marrouche, L. Masetti, F. Meijers, S. Mersi, E. Meschi, F. Moortgat, S. Morovic, M. Mulders, P. Musella, L. Orsini, L. Pape, E. Perez, L. Perrozzi, A. Petrilli, G. Petrucciani, A. Pfeiffer, M. Pierini, M. Pimiä, D. Piparo, M. Plagge, A. Racz, G. Rolandi, M. Rovere, H. Sakulin, C. Schäfer, C. Schwick, A. Sharma, P. Siegrist, P. Silva, M. Simon, P. Sphicas, D. Spiga, J. Steggemann, B. Stieger, M. Stoye, Y. Takahashi, D. Treille, A. Tsirou, G. I. Veres, N. Wardle, H. K. Wöhri, H. Wollny, W. D. Zeuner, W. Bertl, K. Deiters, W. Erdmann, R. Horisberger, Q. Ingram, H. C. Kaestli, D. Kotlinski, U. Langenegger, D. Renker, T. Rohe, F. Bachmair, L. Bäni, L. Bianchini, M. A. Buchmann, B. Casal, N. Chanon, G. Dissertori, M. Dittmar, M. Donegà, M. Dünser, P. Eller, C. Grab, D. Hits, J. Hoss, W. Lustermann, B. Mangano, A. C. Marini, P. Martinez Ruiz del Arbol, M. Masciovecchio, D. Meister, N. Mohr, C. Nägeli, F. Nessi-Tedaldi, F. Pandolfi, F. Pauss, M. Peruzzi, M. Quittnat, L. Rebane, M. Rossini, A. Starodumov, M. Takahashi, K. Theofilatos, R. Wallny, H. A. Weber, C. Amsler, M. F. Canelli, V. Chiochia, A. De Cosa, A. Hinzmann, T. Hreus, B. Kilminster, C. Lange, B. Millan Mejias, J. Ngadiuba, P. Robmann, F. J. Ronga, S. Taroni, M. Verzetti, Y. Yang, M. Cardaci, K. H. Chen, C. Ferro, C. M. Kuo, W. Lin, Y. J. Lu, R. Volpe, S. S. Yu, P. Chang, Y. H. Chang, Y. W. Chang, Y. Chao, K. F. Chen, P. H. Chen, C. Dietz, U. Grundler, W.-S. Hou, K. Y. Kao, Y. J. Lei, Y. F. Liu, R.-S. Lu, D. Majumder, E. Petrakou, Y. M. Tzeng, R. Wilken, B. Asavapibhop, G. Singh, N. Srimanobhas, N. Suwonjandee, A. Adiguzel, M. N. Bakirci, S. Cerci, C. Dozen, I. Dumanoglu, E. Eskut, S. Girgis, G. Gokbulut, E. Gurpinar, I. Hos, E. E. Kangal, A. Kayis Topaksu, G. Onengut, K. Ozdemir, S. Ozturk, A. Polatoz, D. Sunar Cerci, B. Tali, H. Topakli, M. Vergili, I. V. Akin, B. Bilin, S. Bilmis, H. Gamsizkan, B. Isildak, G. Karapinar, K. Ocalan, S. Sekmen, U. E. Surat, M. Yalvac, M. Zeyrek, E. Gülmez, B. Isildak, M. Kaya, O. Kaya, K. Cankocak, F. I. Vardarlı, L. Levchuk, P. Sorokin, J. J. Brooke, E. Clement, D. Cussans, H. Flacher, J. Goldstein, M. Grimes, G. P. Heath, H. F. Heath, J. Jacob, L. Kreczko, C. Lucas, Z. Meng, D. M. Newbold, S. Paramesvaran, A. Poll, S. Senkin, V. J. Smith, T. Williams, K. W. Bell, A. Belyaev, C. Brew, R. M. Brown, D. J. A. Cockerill, J. A. Coughlan, K. Harder, S. Harper, E. Olaiya, D. Petyt, C. H. Shepherd-Themistocleous, A. Thea, I. R. Tomalin, W. J. Womersley, S. D. Worm, M. Baber, R. Bainbridge, O. Buchmuller, D. Burton, D. Colling, N. Cripps, M. Cutajar, P. Dauncey, G. Davies, M. Della Negra, P. Dunne, W. Ferguson, J. Fulcher, D. Futyan, A. Gilbert, G. Hall, G. Iles, M. Jarvis, G. Karapostoli, M. Kenzie, R. Lane, R. Lucas, L. Lyons, A.-M. Magnan, S. Malik, B. Mathias, J. Nash, A. Nikitenko, J. Pela, M. Pesaresi, K. Petridis, D. M. Raymond, S. Rogerson, A. Rose, C. Seez, P. Sharp, A. Tapper, M. Vazquez Acosta, T. Virdee, S. C. Zenz, J. E. Cole, P. R. Hobson, A. Khan, P. Kyberd, D. Leggat, D. Leslie, W. Martin, I. D. Reid, P. Symonds, L. Teodorescu, M. Turner, J. Dittmann, K. Hatakeyama, A. Kasmi, H. Liu, T. Scarborough, O. Charaf, S. I. Cooper, C. Henderson, P. Rumerio, A. Avetisyan, T. Bose, C. Fantasia, P. Lawson, C. Richardson, J. Rohlf, J. St. John, L. Sulak, J. Alimena, E. Berry, S. Bhattacharya, G. Christopher, D. Cutts, Z. Demiragli, N. Dhingra, A. Ferapontov, A. Garabedian, U. Heintz, G. Kukartsev, E. Laird, G. Landsberg, M. Luk, M. Narain, M. Segala, T. Sinthuprasith, T. Speer, J. Swanson, R. Breedon, G. Breto, M. Calderon De La Barca Sanchez, S. Chauhan, M. Chertok, J. Conway, R. Conway, P. T. Cox, R. Erbacher, M. Gardner, W. Ko, R. Lander, T. Miceli, M. Mulhearn, D. Pellett, J. Pilot, F. Ricci-Tam, M. Searle, S. Shalhout, J. Smith, M. Squires, D. Stolp, M. Tripathi, S. Wilbur, R. Yohay, R. Cousins, P. Everaerts, C. Farrell, J. Hauser, M. Ignatenko, G. Rakness, E. Takasugi, V. Valuev, M. Weber, K. Burt, R. Clare, J. Ellison, J. W. Gary, G. Hanson, J. Heilman, M. Ivova Rikova, P. Jandir, E. Kennedy, F. Lacroix, O. R. Long, A. Luthra, M. Malberti, H. Nguyen, M. Olmedo Negrete, A. Shrinivas, S. Sumowidagdo, S. Wimpenny, W. Andrews, J. G. Branson, G. B. Cerati, S. Cittolin, R. T. D’Agnolo, D. Evans, A. Holzner, R. Kelley, D. Klein, M. Lebourgeois, J. Letts, I. Macneill, D. Olivito, S. Padhi, C. Palmer, M. Pieri, M. Sani, V. Sharma, S. Simon, E. Sudano, M. Tadel, Y. Tu, A. Vartak, C. Welke, F. Würthwein, A. Yagil, D. Barge, J. Bradmiller-Feld, C. Campagnari, T. Danielson, A. Dishaw, K. Flowers, M. Franco Sevilla, P. Geffert, C. George, F. Golf, L. Gouskos, J. Incandela, C. Justus, N. Mccoll, J. Richman, D. Stuart, W. To, C. West, J. Yoo, A. Apresyan, A. Bornheim, J. Bunn, Y. Chen, J. Duarte, A. Mott, H. B. Newman, C. Pena, C. Rogan, M. Spiropulu, V. Timciuc, J. R. Vlimant, R. Wilkinson, S. Xie, R. Y. Zhu, V. Azzolini, A. Calamba, B. Carlson, T. Ferguson, Y. Iiyama, M. Paulini, J. Russ, H. Vogel, I. Vorobiev, J. P. Cumalat, W. T. Ford, A. Gaz, E. Luiggi Lopez, U. Nauenberg, J. G. Smith, K. Stenson, K. A. Ulmer, S. R. Wagner, J. Alexander, A. Chatterjee, J. Chu, S. Dittmer, N. Eggert, N. Mirman, G. Nicolas Kaufman, J. R. Patterson, A. Ryd, E. Salvati, L. Skinnari, W. Sun, W. D. Teo, J. Thom, J. Thompson, J. Tucker, Y. Weng, L. Winstrom, P. Wittich, D. Winn, S. Abdullin, M. Albrow, J. Anderson, G. Apollinari, L. A. T. Bauerdick, A. Beretvas, J. Berryhill, P. C. Bhat, G. Bolla, K. Burkett, J. N. Butler, H. W. K. Cheung, F. Chlebana, S. Cihangir, V. D. Elvira, I. Fisk, J. Freeman, Y. Gao, E. Gottschalk, L. Gray, D. Green, S. Grünendahl, O. Gutsche, J. Hanlon, D. Hare, R. M. Harris, J. Hirschauer, B. Hooberman, S. Jindariani, M. Johnson, U. Joshi, K. Kaadze, B. Klima, B. Kreis, S. Kwan, J. Linacre, D. Lincoln, R. Lipton, T. Liu, J. Lykken, K. Maeshima, J. M. Marraffino, V. I. Martinez Outschoorn, S. Maruyama, D. Mason, P. McBride, P. Merkel, K. Mishra, S. Mrenna, Y. Musienko, S. Nahn, C. Newman-Holmes, V. O’Dell, O. Prokofyev, E. Sexton-Kennedy, S. Sharma, A. Soha, W. J. Spalding, L. Spiegel, L. Taylor, S. Tkaczyk, N. V. Tran, L. Uplegger, E. W. Vaandering, R. Vidal, A. Whitbeck, J. Whitmore, F. Yang, D. Acosta, P. Avery, P. Bortignon, D. Bourilkov, M. Carver, T. Cheng, D. Curry, S. Das, M. De Gruttola, G. P. Di Giovanni, R. D. Field, M. Fisher, I. K. Furic, J. Hugon, J. Konigsberg, A. Korytov, T. Kypreos, J. F. Low, K. Matchev, P. Milenovic, G. Mitselmakher, L. Muniz, A. Rinkevicius, L. Shchutska, M. Snowball, D. Sperka, J. Yelton, M. Zakaria, S. Hewamanage, S. Linn, P. Markowitz, G. Martinez, J. L. Rodriguez, T. Adams, A. Askew, J. Bochenek, B. Diamond, J. Haas, S. Hagopian, V. Hagopian, K. F. Johnson, H. Prosper, V. Veeraraghavan, M. Weinberg, M. M. Baarmand, M. Hohlmann, H. Kalakhety, F. Yumiceva, M. R. Adams, L. Apanasevich, V. E. Bazterra, D. Berry, R. R. Betts, I. Bucinskaite, R. Cavanaugh, O. Evdokimov, L. Gauthier, C. E. Gerber, D. J. Hofman, S. Khalatyan, P. Kurt, D. H. Moon, C. O’Brien, C. Silkworth, P. Turner, N. Varelas, E. A. Albayrak, B. Bilki, W. Clarida, K. Dilsiz, F. Duru, M. Haytmyradov, J.-P. Merlo, H. Mermerkaya, A. Mestvirishvili, A. Moeller, J. Nachtman, H. Ogul, Y. Onel, F. Ozok, A. Penzo, R. Rahmat, S. Sen, P. Tan, E. Tiras, J. Wetzel, T. Yetkin, K. Yi, B. A. Barnett, B. Blumenfeld, S. Bolognesi, D. Fehling, A. V. Gritsan, P. Maksimovic, C. Martin, M. Swartz, P. Baringer, A. Bean, G. Benelli, C. Bruner, R. P. Kenny, M. Malek, M. Murray, D. Noonan, S. Sanders, J. Sekaric, R. Stringer, Q. Wang, J. S. Wood, A. F. Barfuss, I. Chakaberia, A. Ivanov, S. Khalil, M. Makouski, Y. Maravin, L. K. Saini, S. Shrestha, N. Skhirtladze, I. Svintradze, J. Gronberg, D. Lange, F. Rebassoo, D. Wright, A. Baden, A. Belloni, B. Calvert, S. C. Eno, J. A. Gomez, N. J. Hadley, R. G. Kellogg, T. Kolberg, Y. Lu, M. Marionneau, A. C. Mignerey, K. Pedro, A. Skuja, M. B. Tonjes, S. C. Tonwar, A. Apyan, R. Barbieri, G. Bauer, W. Busza, I. A. Cali, M. Chan, L. Di Matteo, V. Dutta, G. Gomez Ceballos, M. Goncharov, D. Gulhan, M. Klute, Y. S. Lai, Y.-J. Lee, A. Levin, P. D. Luckey, T. Ma, C. Paus, D. Ralph, C. Roland, G. Roland, G. S. F. Stephans, F. Stöckli, K. Sumorok, D. Velicanu, J. Veverka, B. Wyslouch, M. Yang, M. Zanetti, V. Zhukova, B. Dahmes, A. Gude, S. C. Kao, K. Klapoetke, Y. Kubota, J. Mans, N. Pastika, R. Rusack, A. Singovsky, N. Tambe, J. Turkewitz, J. G. Acosta, S. Oliveros, E. Avdeeva, K. Bloom, S. Bose, D. R. Claes, A. Dominguez, R. Gonzalez Suarez, J. Keller, D. Knowlton, I. Kravchenko, J. Lazo-Flores, S. Malik, F. Meier, G. R. Snow, M. Zvada, J. Dolen, A. Godshalk, I. Iashvili, A. Kharchilava, A. Kumar, S. Rappoccio, G. Alverson, E. Barberis, D. Baumgartel, M. Chasco, J. Haley, A. Massironi, D. M. Morse, D. Nash, T. Orimoto, D. Trocino, R. J. Wang, D. Wood, J. Zhang, K. A. Hahn, A. Kubik, N. Mucia, N. Odell, B. Pollack, A. Pozdnyakov, M. Schmitt, S. Stoynev, K. Sung, M. Velasco, S. Won, A. Brinkerhoff, K. M. Chan, A. Drozdetskiy, M. Hildreth, C. Jessop, D. J. Karmgard, N. Kellams, K. Lannon, W. Luo, S. Lynch, N. Marinelli, T. Pearson, M. Planer, R. Ruchti, N. Valls, M. Wayne, M. Wolf, A. Woodard, L. Antonelli, J. Brinson, B. Bylsma, L. S. Durkin, S. Flowers, C. Hill, R. Hughes, K. Kotov, T. Y. Ling, D. Puigh, M. Rodenburg, G. Smith, B. L. Winer, H. Wolfe, H. W. Wulsin, O. Driga, P. Elmer, P. Hebda, A. Hunt, S. A. Koay, P. Lujan, D. Marlow, T. Medvedeva, M. Mooney, J. Olsen, P. Piroué, X. Quan, H. Saka, D. Stickland, C. Tully, J. S. Werner, A. Zuranski, E. Brownson, H. Mendez, J. E. Ramirez Vargas, V. E. Barnes, D. Benedetti, D. Bortoletto, M. De Mattia, L. Gutay, Z. Hu, M. K. Jha, M. Jones, K. Jung, M. Kress, N. Leonardo, D. Lopes Pegna, V. Maroussov, D. H. Miller, N. Neumeister, B. C. Radburn-Smith, X. Shi, I. Shipsey, D. Silvers, A. Svyatkovskiy, F. Wang, W. Xie, L. Xu, H. D. Yoo, J. Zablocki, Y. Zheng, N. Parashar, J. Stupak, A. Adair, B. Akgun, K. M. Ecklund, F. J. M. Geurts, W. Li, B. Michlin, B. P. Padley, R. Redjimi, J. Roberts, J. Zabel, B. Betchart, A. Bodek, R. Covarelli, P. de Barbaro, R. Demina, Y. Eshaq, T. Ferbel, A. Garcia-Bellido, P. Goldenzweig, J. Han, A. Harel, A. Khukhunaishvili, G. Petrillo, D. Vishnevskiy, R. Ciesielski, L. Demortier, K. Goulianos, G. Lungu, C. Mesropian, S. Arora, A. Barker, J. P. Chou, C. Contreras-Campana, E. Contreras-Campana, D. Duggan, D. Ferencek, Y. Gershtein, R. Gray, E. Halkiadakis, D. Hidas, S. Kaplan, A. Lath, S. Panwalkar, M. Park, R. Patel, S. Salur, S. Schnetzer, S. Somalwar, R. Stone, S. Thomas, P. Thomassen, M. Walker, K. Rose, S. Spanier, A. York, O. Bouhali, A. Castaneda Hernandez, R. Eusebi, W. Flanagan, J. Gilmore, T. Kamon, V. Khotilovich, V. Krutelyov, R. Montalvo, I. Osipenkov, Y. Pakhotin, A. Perloff, J. Roe, A. Rose, A. Safonov, T. Sakuma, I. Suarez, A. Tatarinov, N. Akchurin, C. Cowden, J. Damgov, C. Dragoiu, P. R. Dudero, J. Faulkner, K. Kovitanggoon, S. Kunori, S. W. Lee, T. Libeiro, I. Volobouev, E. Appelt, A. G. Delannoy, S. Greene, A. Gurrola, W. Johns, C. Maguire, Y. Mao, A. Melo, M. Sharma, P. Sheldon, B. Snook, S. Tuo, J. Velkovska, M. W. Arenton, S. Boutle, B. Cox, B. Francis, J. Goodell, R. Hirosky, A. Ledovskoy, H. Li, C. Lin, C. Neu, J. Wood, C. Clarke, R. Harr, P. E. Karchin, C. Kottachchi Kankanamge Don, P. Lamichhane, J. Sturdy, D. A. Belknap, D. Carlsmith, M. Cepeda, S. Dasu, L. Dodd, S. Duric, E. Friis, R. Hall-Wilton, M. Herndon, A. Hervé, P. Klabbers, A. Lanaro, C. Lazaridis, A. Levine, R. Loveless, A. Mohapatra, I. Ojalvo, T. Perry, G. A. Pierro, G. Polese, I. Ross, T. Sarangi, A. Savin, W. H. Smith, D. Taylor, P. Verwilligen, C. Vuosalo, N. Woods, [Authorinst]The CMS Collaboration

**Affiliations:** Yerevan Physics Institute, Yerevan, Armenia; Institut für Hochenergiephysik der OeAW, wien, Austria; National Centre for Particle and High Energy Physics, Minsk, Belarus; Universiteit Antwerpen, Antwerpen, Belgium; Vrije Universiteit Brussel, Brussels, Belgium; Université Libre de Bruxelles, Bruxelles, Belgium; Ghent University, Ghent, Belgium; Université Catholique de Louvain, Louvain-la-Neuve, Belgium; Université de Mons, Mons, Belgium; Centro Brasileiro de Pesquisas Fisicas, Rio de Janeiro, Brazil; Universidade do Estado do Rio de Janeiro, Rio de Janeiro, Brazil; Universidade Estadual Paulista, Universidade Federal do ABC, São Paulo, Brazil; Institute for Nuclear Research and Nuclear Energy, Sofia, Bulgaria; University of Sofia, Sofia, Bulgaria; Institute of High Energy Physics, Beijing, China; State Key Laboratory of Nuclear Physics and Technology, Peking University, Beijing, China; Universidad de Los Andes, Bogota, Colombia; Faculty of Electrical Engineering, Mechanical Engineering and Naval Architecture, University of Split, Split, Croatia; Faculty of Science, University of Split, Split, Croatia; Institute Rudjer Boskovic, Zagreb, Croatia; University of Cyprus, Nicosia, Cyprus; Charles University, Prague, Czech Republic; Academy of Scientific Research and Technology of the Arab Republic of Egypt, Egyptian Network of High Energy Physics, Cairo, Egypt; National Institute of Chemical Physics and Biophysics, Tallinn, Estonia; Department of Physics, University of Helsinki, Helsinki, Finland; Helsinki Institute of Physics, Helsinki, Finland; Lappeenranta University of Technology, Lappeenranta, Finland; DSM/IRFU, CEA/Saclay, Gif-sur-Yvette, France; Laboratoire Leprince-Ringuet, Ecole Polytechnique, IN2P3-CNRS, Palaiseau, France; Institut Pluridisciplinaire Hubert Curien, Université de Strasbourg, Université de Haute Alsace Mulhouse, CNRS/IN2P3, Strasbourg, France; Centre de Calcul de l’Institut National de Physique Nucleaire et de Physique des Particules, CNRS/IN2P3, Villeurbanne, France; Institut de Physique Nucléaire de Lyon, Université de Lyon, Université Claude Bernard Lyon 1, CNRS-IN2P3, Villeurbanne, France; Institute of High Energy Physics and Informatization, Tbilisi State University, Tbilisi, Georgia; I. Physikalisches Institut, RWTH Aachen University, Aachen, Germany; III. Physikalisches Institut A, RWTH Aachen University, Aachen, Germany; III. Physikalisches Institut B, RWTH Aachen University, Aachen, Germany; Deutsches Elektronen-Synchrotron, Hamburg, Germany; University of Hamburg, Hamburg, Germany; Institut für Experimentelle Kernphysik, Karlsruhe, Germany; Institute of Nuclear and Particle Physics (INPP), NCSR Demokritos, Aghia Paraskevi, Greece; University of Athens, Athens, Greece; University of Ioánnina, Ioánnina, Greece; Wigner Research Centre for Physics, Budapest, Hungary; Institute of Nuclear Research ATOMKI, Debrecen, Hungary; University of Debrecen, Debrecen, Hungary; National Institute of Science Education and Research, Bhubaneswar, India; Panjab University, Chandigarh, India; University of Delhi, Delhi, India; Saha Institute of Nuclear Physics, Kolkata, India; Bhabha Atomic Research Centre, Mumbai, India; Tata Institute of Fundamental Research, Mumbai, India; Institute for Research in Fundamental Sciences (IPM), Tehran, Iran; University College Dublin, Dublin, Ireland; INFN Sezione di Bari, Università di Bari, Politecnico di Bari, Bari, Italy; INFN Sezione di Bologna, Università di Bologna, Bologna, Italy; INFN Sezione di Catania, Università di Catania, CSFNSM, Catania, Italy; INFN Sezione di Firenze, Università di Firenze, Firenze, Italy; INFN Laboratori Nazionali di Frascati, Frascati, Italy; INFN Sezione di Genova, Università di Genova, Genova, Italy; INFN Sezione di Milano-Bicocca, Università di Milano-Bicocca, Milano, Italy; INFN Sezione di Napoli, Università di Napoli ’Federico II’, Università della Basilicata (Potenza), Università G. Marconi (Roma), Naples, Italy; INFN Sezione di Padova, Università di Padova, Università di Trento (Trento), Padua, Italy; INFN Sezione di Pavia, Università di Pavia, Pavia, Italy; INFN Sezione di Perugia, Università di Perugia, Perugia, Italy; INFN Sezione di Pisa, Università di Pisa, Scuola Normale Superiore di Pisa, Pisa, Italy; INFN Sezione di Roma, Università di Roma, Rome, Italy; INFN Sezione di Torino, Università di Torino, Università del Piemonte Orientale (Novara), Torino, Italy; INFN Sezione di Trieste, Università di Trieste, Trieste, Italy; Kangwon National University, Chunchon, Korea; Kyungpook National University, Taegu, Korea; Chonbuk National University, Chonju, Korea; Chonnam National University, Institute for Universe and Elementary Particles, Kwangju, Korea; Korea University, Seoul, Korea; University of Seoul, Seoul, Korea; Sungkyunkwan University, Suwon, Korea; Vilnius University, Vilnius, Lithuania; National Centre for Particle Physics, Universiti Malaya, Kuala Lumpur, Malaysia; Centro de Investigacion y de Estudios Avanzados del IPN, Mexico City, Mexico; Universidad Iberoamericana, Mexico City, Mexico; Benemerita Universidad Autonoma de Puebla, Puebla, Mexico; Universidad Autónoma de San Luis Potosí, San Luis Potosí, Mexico; University of Auckland, Auckland, New Zealand; University of Canterbury, Christchurch, New Zealand; National Centre for Physics, Quaid-I-Azam University, Islamabad, Pakistan; National Centre for Nuclear Research, Swierk, Poland; Institute of Experimental Physics, Faculty of Physics, University of Warsaw, Warsaw, Poland; Laboratório de Instrumentação e Física Experimental de Partículas, Lisbon, Portugal; Joint Institute for Nuclear Research, Dubna, Russia; Petersburg Nuclear Physics Institute, Gatchina, St. Petersburg, Russia; Institute for Nuclear Research, Moscow, Russia; Institute for Theoretical and Experimental Physics, Moscow, Russia; P. N. Lebedev Physical Institute, Moscow, Russia; Skobeltsyn Institute of Nuclear Physics, Lomonosov Moscow State University, Moscow, Russia; State Research Center of Russian Federation, Institute for High Energy Physics, Protvino, Russia; Faculty of Physics and Vinca Institute of Nuclear Sciences, University of Belgrade, Belgrade, Serbia; Centro de Investigaciones Energéticas Medioambientales y Tecnológicas (CIEMAT), Madrid, Spain; Universidad Autónoma de Madrid, Madrid, Spain; Universidad de Oviedo, Oviedo, Spain; Instituto de Física de Cantabria (IFCA), CSIC-Universidad de Cantabria, Santander, Spain; CERN, European Organization for Nuclear Research, Geneva, Switzerland; Paul Scherrer Institut, Villigen, Switzerland; Institute for Particle Physics, ETH Zurich, Zurich, Switzerland; Universität Zürich, Zurich, Switzerland; National Central University, Chung-Li, Taiwan; National Taiwan University (NTU), Taipei, Taiwan; Department of Physics, Faculty of Science, Chulalongkorn University, Bangkok, Thailand; Cukurova University, Adana, Turkey; Physics Department, Middle East Technical University, Ankara, Turkey; Bogazici University, Istanbul, Turkey; Istanbul Technical University, Istanbul, Turkey; National Scientific Center, Kharkov Institute of Physics and Technology, Kharkov, Ukraine; University of Bristol, Bristol, UK; Rutherford Appleton Laboratory, Didcot, UK; Imperial College, London, UK; Brunel University, Uxbridge, UK; Baylor University, Waco, USA; The University of Alabama, Tuscaloosa, USA; Boston University, Boston, USA; Brown University, Providence, USA; University of California, Davis, USA; University of California, Los Angeles, USA; University of California, Riverside, Riverside, USA; University of California, San Diego, La Jolla, USA; University of California, Santa Barbara, Santa Barbara USA; California Institute of Technology, Pasadena, USA; Carnegie Mellon University, Pittsburgh, USA; University of Colorado at Boulder, Boulder, USA; Cornell University, Ithaca, USA; Fairfield University, Fairfield, USA; Fermi National Accelerator Laboratory, Batavia, USA; University of Florida, Gainesville, USA; Florida International University, Miami, USA; Florida State University, Tallahassee, USA; Florida Institute of Technology, Melbourne, USA; University of Illinois at Chicago (UIC), Chicago, USA; The University of Iowa, Iowa City, USA; Johns Hopkins University, Baltimore, USA; The University of Kansas, Lawrence, USA; Kansas State University, Manhattan, USA; Lawrence Livermore National Laboratory, Livermore, USA; University of Maryland, College Park, USA; Massachusetts Institute of Technology, Cambridge, USA; University of Minnesota, Minneapolis, USA; University of Mississippi, Oxford, USA; University of Nebraska-Lincoln, Lincoln, USA; State University of New York at Buffalo, Buffalo, USA; Northeastern University, Boston, USA; Northwestern University, Evanston, USA; University of Notre Dame, Notre Dame, USA; The Ohio State University, Columbus, USA; Princeton University, Princeton, USA; University of Puerto Rico, Mayaguez, USA; Purdue University, West Lafayette, USA; Purdue University Calumet, Hammond, USA; Rice University, Houston, USA; University of Rochester, Rochester, USA; The Rockefeller University, New York, USA; Rutgers, The State University of New Jersey, Piscataway, USA; University of Tennessee, Knoxville, USA; Texas A&M University, College Station, USA; Texas Tech University, Lubbock, USA; Vanderbilt University, Nashville, USA; University of Virginia, Charlottesville, USA; Wayne State University, Detroit, USA; University of Wisconsin, Madison, USA; CERN, Geneva, Switzerland

**Keywords:** CMS, Physics, QCD, Jets, 3-jet mass, PDF, Strong coupling constant, Alpha-S

## Abstract

This paper presents a measurement of the inclusive 3-jet production differential cross section at a proton–proton centre-of-mass energy of 7 TeV using data corresponding to an integrated luminosity of 5$\,\mathrm{fb}^{-1}$collected with the CMS detector. The analysis is based on the three jets with the highest transverse momenta. The cross section is measured as a function of the invariant mass of the three jets in a range of 445–3270 GeV and in two bins of the maximum rapidity of the jets up to a value of 2. A comparison between the measurement and the prediction from perturbative QCD at next-to-leading order is performed. Within uncertainties, data and theory are in agreement. The sensitivity of the observable to the strong coupling constant $\alpha _\mathrm {S}$ is studied. A fit to all data points with 3-jet masses larger than 664 GeV gives a value of the strong coupling constant of $\alpha _S(M_\mathrm{Z}) = 0.1171 \pm 0.0013\,\text {(exp)} \,^{+0.0073}_{-0.0047}\,\text {(theo)}$.

## Introduction

A key characteristic of highly energetic proton–proton collisions at the LHC is the abundant production of multijet events. At high transverse momenta $p_{\mathrm {T}}$, such events are described by quantum chromodynamics (QCD) in terms of parton–parton scattering. The simplest jet production process corresponds to a $2 \rightarrow 2$ reaction with the two outgoing partons fragmenting into a pair of jets. Two cross sections, for which the leading-order (LO) predictions in perturbative QCD (pQCD) are proportional to the square of the strong coupling constant, $\alpha _\mathrm {S} ^2$, are conventionally defined: the inclusive single-jet cross section as a function of jet $p_{\mathrm {T}}$ and rapidity $y$, and the 2-jet production cross section as a function of the 2-jet invariant mass and a rapidity-related kinematic quantity that provides a separation of the phase space into exclusive bins. The ATLAS Collaboration usually characterizes the 2-jet system in terms of the rapidity separation of the two jets leading in $p_{\mathrm {T}}$, while CMS employs the larger of the two absolute rapidities of the two jets. Corresponding measurements by the ATLAS and CMS Collaborations can be found in Refs. [1–6].

In this paper, the inclusive 3-jet production differential cross section is measured as a function of the invariant mass $m_3$ of the three jets leading in $p_{\mathrm {T}}$ and of their maximum rapidity $y_{\text {max}}$, which are defined as follows:

1 $$\begin{aligned} m_3 ^{2}= & {} \left( p_{1}+p_{2}+p_{3}\right) ^{2} \nonumber \\ y_{\text {max}}= & {} {{\mathrm{sgn}}}\big ( |\max (y_1, y_2, y_3) | \nonumber \\&- |\min (y_1, y_2, y_3) |\big ) \cdot \max \left( |y_1 |, |y_2 |, |y_3 | \right) , \end{aligned}$$

2 $$\begin{aligned} m_3 ^{2}= & {} \left( p_{1}+p_{2}+p_{3}\right) ^{2} \nonumber \\ y_{\text {max}}= & {} {{\mathrm{sgn}}}\big (|\max (y_1, y_2, y_3) | - |\min (y_1, y_2, y_3) |\big ) \nonumber \\&\cdot \max \left( |y_1 |, |y_2 |, |y_3 | \right) , \end{aligned}$$

where $p_i$ and $y_i$ are the four-momentum and rapidity of the $i$th jet leading in $p_{\mathrm {T}}$. Following Ref. [3], $y_{\text {max}}$ is defined as a signed quantity such that the double-differential cross section, $\mathrm{d}^2\sigma /\mathrm{d}{m_3}\,\mathrm{d}{y_{\text {max}}}$, can be written in a way similar to the inclusive jet cross section, $\mathrm{d}^2\sigma /\mathrm{d}{p_{\mathrm {T}}}\,\mathrm{d}{y}$, including a factor of 2 for rapidity bin widths in terms of $|y_{\text {max}} |$ and $|y |$, respectively. The absolute value of $y_{\text {max}}$ is equal to the maximum $|y |$ of the jets, denoted $| y |_{\text {max}}$. A previous study of the 3-jet mass spectra was published by the D0 Collaboration [7]. Very recently, ATLAS submitted a 3-jet cross section measurement [8].

For this cross section, the LO process is proportional to $\alpha _\mathrm {S} ^3$ and theoretical predictions are available up to next-to-leading order (NLO) [9, 10] making precise comparisons to data possible. The potential impact of this measurement on the parton distribution functions (PDFs) of the proton is studied and the strong coupling constant $\alpha _\mathrm {S}$ is extracted. In previous publications by CMS, the value of $\alpha _\mathrm {S}$ was determined to $\alpha _S(M_\mathrm{Z}) = 0.1148 \pm 0.0014\,\text {(exp)} \pm 0.0050\,\text {(theo)}$ by investigating the ratio of inclusive 3-jet to inclusive 2-jet production, $R_\mathrm {32}$  [11], and $\alpha _S(M_\mathrm{Z}) = 0.1185 \pm 0.0019\,\text {(exp)} \,^{+0.0060}_{-0.0037}\,\text {(theo)}$ by fitting the inclusive jet cross section [12]. The ratio $R_\mathrm {32}$ benefits from uncertainty cancellations, but it is only proportional to $\alpha _\mathrm {S}$ at LO, leading to a correspondingly high sensitivity to its experimental uncertainties in fits of $\alpha _S(M_\mathrm{Z})$. The second observable, which is similar to the denominator in $R_\mathrm {32}$, is proportional to $\alpha _\mathrm {S} ^2$ at LO with a sensitivity to experimental uncertainties reduced by a factor of $1/2$, but without uncertainty cancellations. It is interesting to study how fits of $\alpha _\mathrm {S}$ to the inclusive 3-jet mass cross section, $\mathrm{d}^2\sigma /\mathrm{d}{m_3}\,\mathrm{d}{y_{\text {max}}}$, which is a 3-jet observable similar to the numerator of $R_\mathrm {32}$, compare to previous results.

The data analyzed in the following were recorded by the CMS detector at the LHC during the 2011 data-taking period at a proton–proton centre-of-mass energy of 7 TeV and correspond to an integrated luminosity of 5.0$\,\mathrm{fb}^{-1}$. Jets are clustered by using the infrared- and collinear-safe anti-$k_{\mathrm {T}}$ algorithm [13] as implemented in the FastJet package [14] with a jet size parameter of $R=0.7$. A smaller jet size parameter of $R=0.5$ has been investigated, but was found to describe the data less well. Similarly, in Ref. [15] it is shown that the inclusive jet cross section is better described by NLO theory for $R=0.7$ than for $R=0.5$.

Events are studied in which at least three jets are found up to a rapidity of $|y |=3$ that are above a minimal $p_{\mathrm {T}}$ threshold of 100 GeV. The jet yields are corrected for detector effects resulting in a final measurement phase space of $445\hbox { GeV}\le m_3 < 3270\hbox { GeV}$ and $| y |_{\text {max}} < 2$. Extension of the analysis to larger values of $| y |_{\text {max}}$ was not feasible with the available trigger paths.

This paper is divided into seven parts. Section 2 presents an overview of the CMS detector and the event reconstruction. Sections 3 and 4 discuss the event selection and present the measurement. Theoretical ingredients are introduced in Sect. 5 and are applied in Sect. 6 to determine $\alpha _S(M_\mathrm{Z})$ from a fit to the measured 3-jet production cross section. Conclusions are presented in Sect. 7.

## Apparatus and event reconstruction

The central feature of the CMS apparatus is a superconducting solenoid of 6 m internal diameter, providing a magnetic field of 3.8 T. Within the superconducting solenoid volume are a silicon pixel and strip tracker, a lead tungstate crystal electromagnetic calorimeter (ECAL), and a brass and scintillator hadron calorimeter (HCAL), each composed of a barrel and two endcap sections. Muons are measured in gas-ionization detectors embedded in the steel flux-return yoke outside the solenoid. Extensive forward calorimetry complements the coverage provided by the barrel and endcap detectors.

The first level (L1) of the CMS trigger system, composed of custom hardware processors, uses information from the calorimeters and muon detectors to select the most interesting events in a fixed time interval of less than 4$\,\mu \text {s}$. The high level trigger (HLT) processor farm further decreases the event rate from around 100 kHz to around 400 Hz, before data storage.

The particle-flow algorithm reconstructs and identifies each particle candidate with an optimized combination of all subdetector information [16, 17]. For each event, the reconstructed particle candidates are clustered into hadronic jets by using the anti-$k_{\mathrm {T}}$ algorithm with a jet size parameter of $R=0.7$. The jet momentum is determined as the vectorial sum of all constituent momenta in this jet, and is found in the simulation to be within 5–10 % of the true momentum over the whole $p_{\mathrm {T}}$ spectrum and detector acceptance. An offset correction is applied to take into account the extra energy clustered into jets due to additional proton–proton interactions within the same or neighbouring bunch crossings (pileup). Jet energy corrections are derived from the simulation, and are confirmed with in situ measurements with the energy balance of dijet, photon+jet, and $\mathrm{Z}$+jet events [18, 19]. The jet energy resolution amounts typically to 15 % at 10 GeV, 8 % at 100 GeV, and 4 % at 1 TeV. A more detailed description of the CMS apparatus can be found in Ref. [20].

## Event selection

The data set used for this analysis contains all events that were triggered by any of the single-jet triggers. A single-jet trigger accepts events if at least one reconstructed jet surpasses a transverse momentum threshold. During the 2011 data-taking period, triggers with eight different thresholds ranging from 60 to 370 GeV were employed. They are listed in Table 1 with the number of events recorded by each trigger and the corresponding turn-on threshold $p_\mathrm {T,99\,\%}$, where the trigger is more than 99 % efficient.

**Table 1:** Trigger and turn-on thresholds in leading jet $p_{\mathrm {T}}$, and the number of events recorded via the single-jet trigger paths used for this measurement

Trigger threshold	Turn-on threshold	Recorded events
$p_{\mathrm {T}}$ ($\text {GeV}$)	$p_\mathrm {T,99\,\%}$ ($\text {GeV}$ )	
$$ 60$$	$$85$$	2 591 154
$$ 80$$	$$110$$	1 491 011
$110$	$144$	2 574 451
$150$	$192$	2 572 083
$190$	$238$	3 533 874
$240$	$294$	3 629 577
$300$	$355$	9 785 529
$370$	$435$	3 129 458

The different triggers are used to measure the 3-jet mass spectrum in mutually exclusive regions of the phase space, defined in terms of the $p_{\mathrm {T}}$ of the leading jet: the $p_{\mathrm {T}}$ interval covered by a single-jet trigger starts at the corresponding turn-on threshold $p_\mathrm {T,99\,\%}$ and ends at the turn-on threshold of the trigger with the next highest threshold. The final 3-jet mass spectrum is obtained by summing the spectra measured with the different triggers while taking trigger prescale factors into account. Apart from the prescaling, the trigger efficiency is more than 99 % across the entire mass range studied.

In the inner rapidity region, most single-jet triggers contribute up to 50 % of the final event yield, with the exception of the two triggers with the lowest and highest threshold, which contribute up to 80 and 100 % respectively, depending on $$m_3 $$. In particular, starting at 1100 GeV, the majority of the events are taken from the highest unprescaled trigger. In the outer rapidity region, each jet trigger contributes over a large range of three-jet masses to the measurement. With the exception of the two triggers with the lowest and highest thresholds, each trigger contributes around 25 % to the final event yield.

The recorded events are filtered with tracking-based selections [21] to remove interactions between the circulating proton bunches and residual gas particles or the beam collimators. To further reject beam backgrounds and off-centre parasitic bunch crossings, standard vertex selection cuts are applied [21]. To enhance the QCD event purity, events in which the missing transverse energy $E_{\mathrm {T}}^{\text {miss}}$ amounts to more than 30 % of the measured total transverse energy are removed. The missing transverse energy is calculated by requiring momentum conservation for the reconstructed particle flow candidates [19].

Jet identification (jet ID) selection criteria [22] are developed to reject pure noise or noise enhanced jets, while keeping more than 99 % of physical jets with transverse momentum above 10 GeV. In contrast to the previous selection criteria, which reject complete events, the jet ID removes only individual jets from the event. The jet ID applied to the particle-flow jets requires that each jet should contain at least two particles, one of which is a charged hadron. In addition, the jet energy fraction carried by neutral hadrons and photons must be less than 90 %. These criteria have an efficiency greater than 99 % for hadronic jets.

## Measurement and experimental uncertainties

The double-differential 3-jet production cross section is measured as a function of the invariant 3-jet mass $m_3$ and the maximum rapidity $y_{\text {max}}$ of the three jets with the highest transverse momenta in the event:

3 $$\begin{aligned} \frac{\mathrm{d}^{2}\sigma }{\mathrm{d}{m_3}\,\mathrm{d}y_{\text {max}}} = \frac{1}{\epsilon \mathcal {L}} \frac{N}{\Delta m_3 (2\Delta | y |_{\text {max}})}. \end{aligned}$$

Here, $\mathcal {L}$ is the integrated luminosity and $N$ is the number of events. The efficiency $\epsilon$ is the product of the trigger and event selection efficiencies, and differs from unity by less than one percent for this jet analysis. Differences in the efficiency with respect to unity are included in a systematic uncertainty. The width of a 3-jet mass bin is based on the 3-jet mass resolution, which is derived from a detector simulation. Starting at $m_3 = 50\hbox { GeV}$, the bin width increases progressively with $m_3$. In addition, the phase space is split into an inner, $| y |_{\text {max}} < 1$, and an outer, $1 \le | y |_{\text {max}} < 2$, rapidity region. The bin widths in $y_{\text {max}}$ are equal to 2. Events with $| y |_{\text {max}} \ge 2$ are rejected.

To remove the impact of detector effects from limited acceptance and finite resolution, the measurement is corrected with the iterative d’Agostini unfolding algorithm [23] with four iterations. Response matrices for the unfolding algorithm are derived from detector simulation by using the two event generators pythia version 6.4.22 [24] with tune Z2 [25] and herwig++ version 2.4.2 [26] with the default tune. (The pythia 6 Z2 tune is identical to the Z1 tune described in [25] except that Z2 uses the CTEQ6L PDF while Z1 uses CTEQ5L.) Differences in the unfolding result are used to evaluate the uncertainties related to assumptions in modelling the parton showering [27, 28], hadronization [29–32], and the underlying event [27, 33, 34] in these event generators. Additional uncertainties are determined from an ensemble of Monte Carlo (MC) experiments, where the data input and the response matrix are varied within the limits of their statistical precision before entering the unfolding algorithm. The unfolding result corresponds to the sample mean, while the statistical uncertainty, which is propagated through the unfolding procedure, is given by the sample covariance. The variation of the input data leads to the statistical uncertainty in the unfolded cross section, while the variation of the response matrix is an additional uncertainty inherent in the unfolding technique because of the limited size of simulated samples.

The systematic uncertainty related to the determination of the jet energy scale (JES) is evaluated via 16 independent sources as described in Ref. [3]. The modified prescription for the treatment of correlations as recommended in Ref. [12] is applied. To reduce artifacts caused by trigger turn-ons and prescale weights, the JES uncertainty is propagated to the cross section measurement by employing an ensemble of MC experiments, where the data input is varied within the limits of the systematic uncertainty and where average prescale weights are used.

The luminosity uncertainty, which is fully correlated across all $m_3$ and $y_{\text {max}}$ bins, is estimated to be 2.2 % [35].

Residual jet reconstruction and trigger inefficiencies are accounted for by an additional uncorrelated uncertainty of 1 % as in Ref. [3].

**Fig. 1:**
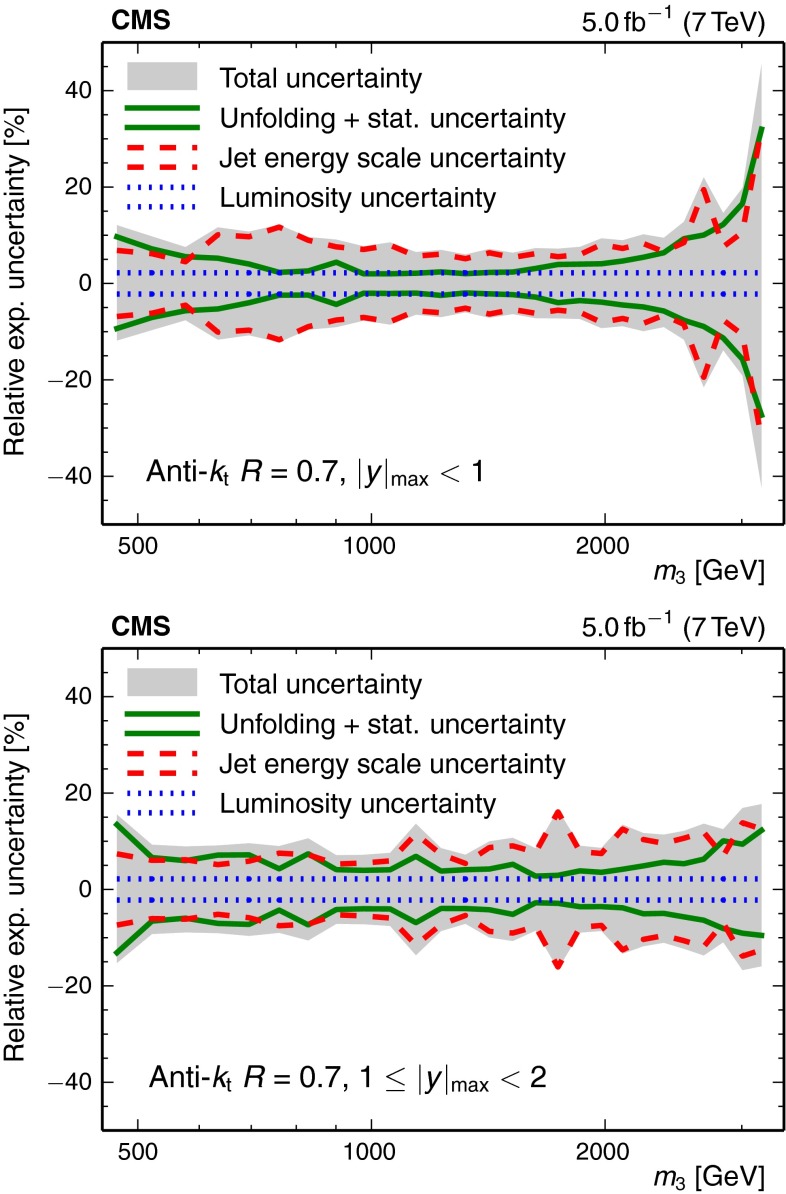
Overview of the measurement uncertainties in the inner $| y |_{\text {max}} < 1$ (*top*) and the outer rapidity region $1 \le | y |_{\text {max}} < 2$ (*bottom*). All uncertainty components, including the 1 % uncorrelated residual uncertainty, are added in quadrature to give the total uncertainty

Figure 1 presents an overview of the experimental uncertainties for the 3-jet mass measurement. Over a wide range of 3-jet masses, the JES uncertainty represents the largest contribution. At the edges of the investigated phase space, i.e. in the low and high 3-jet mass regions, statistical and unfolding uncertainties, which are intrinsically linked through the unfolding procedure, become major contributors to the total uncertainty.

## Theoretical predictions and uncertainties

The theoretical predictions for the 3-jet mass cross sections consist of an NLO QCD calculation and a nonperturbative (NP) correction to account for the underlying event modelled by multiparton interactions (MPI) and for hadronization effects. Electroweak corrections to inclusive and dijet cross sections have been calculated in Ref. [36], where they are found to be limited to a few percent at the highest dijet masses accessible with the CMS data at 7 TeV centre-of-mass energy. For 3-jet quantities these corrections are not known and hence cannot be considered in the present analysis.

The NLO calculations are performed by using the NLOJet++ program version 4.1.3 [9, 10] within the framework of the fastNLO package version 2.1 [37]. The partonic events are subjected to the same jet algorithm and phase space selections as the data events, where at least three jets with $|y |\le 3$ and $p_{\mathrm {T}} > 100\hbox { GeV}$ are required. The number of massless quark flavours, $N_f$, is set to five. The impact of jet production via massive top-antitop quark pairs is estimated to be negligible. The renormalization and factorization scales, $\mu _r$ and $\mu _f$, are identified with $m_3/2$. With this choice, which is identical to the jet $p_{\mathrm {T}}$ in case of dijet events at central rapidity with $m_2/2$ as scale, the NLO corrections to the LO cross sections remain limited between 1.2 and 1.6. The uncertainty in the predicted cross section associated with the renormalization and factorization scale choice is evaluated by varying $\mu _r$ and $\mu _f$ from the default by the following six combinations: $(\mu _r/(m_3/2),\mu _f/(m_3/2)) = (1/2,1/2)$, $(1/2,1)$, $(1,1/2)$, $(1,2)$, $(2,1)$, and $(2,2)$.

Comparisons to the NLO predictions are performed for five different PDF sets, each with NLO and NNLO PDF evolutions, from the LHAPDF package [38]. They are listed in Table 2 together with the corresponding number of active flavours, $N_f$, the default values of the strong coupling constant $\alpha _S(M_\mathrm{Z})$, and the ranges in $\alpha _S(M_\mathrm{Z})$ available for fits. All PDF sets include a maximum of five active flavours $N_f$ except for NNPDF2.1, which has $N_{f,\text {max}} = 6$. Only the ABM11 PDF set employs a fixed-flavour number scheme in contrast to variable-flavour number schemes favoured by all other PDF sets. The PDF uncertainties in the cross section predictions are evaluated according to the prescriptions recommended for the respective PDFs. More details are available in the references listed in Table 2.

**Table 2:** The PDF sets used in comparisons to the data together with the evolution order (Evol.), the corresponding number of active flavours, $N_f$, the assumed masses $M_\mathrm{t}$ and $M_\mathrm{Z}$ of the top quark and the $\mathrm{Z}$ boson, respectively, the default values of $\alpha _S(M_\mathrm{Z})$, and the range in $\alpha _S(M_\mathrm{Z})$ variation available for fits. For CT10 the updated versions of 2012 are taken

Base set	Refs.	Evol.	$N_f$	$M_\mathrm{t}$ ($\text {GeV}$)	$M_\mathrm{Z}$ ($\text {GeV}$)	$\alpha _S(M_\mathrm{Z})$	$\alpha _S(M_\mathrm{Z})$ range
ABM11	[39]	NLO	5	180	91.174	0.1180	0.110–0.130
ABM11	[39]	NNLO	5	180	91.174	0.1134	0.104–0.120
CT10	[40]	NLO	$\le$5	172	91.188	0.1180	0.112–0.127
CT10	[40]	NNLO	$\le$5	172	91.188	0.1180	0.110–0.130
HERAPDF1.5	[41]	NLO	$\le$5	180	91.187	0.1176	0.114–0.122
HERAPDF1.5	[41]	NNLO	$\le$5	180	91.187	0.1176	0.114–0.122
MSTW2008	[42, 43]	NLO	$\le$5	$10^{10}$	91.1876	0.1202	0.110–0.130
MSTW2008	[42, 43]	NNLO	$\le$5	$10^{10}$	91.1876	0.1171	0.107–0.127
NNPDF2.1	[44]	NLO	$\le$6	175	91.2	0.1190	0.114–0.124
NNPDF2.1	[44]	NNLO	$\le$6	175	91.2	0.1190	0.114–0.124

**Fig. 2:**
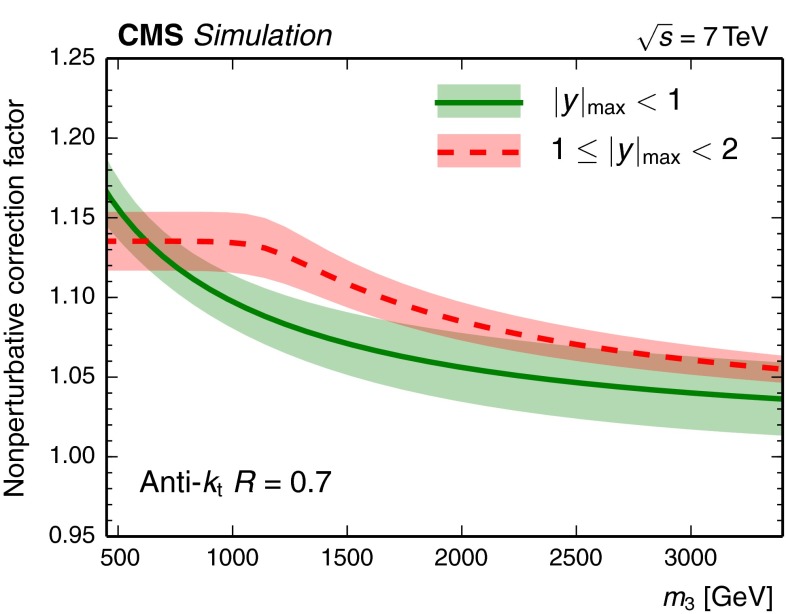
Overview of the NP correction factors and their uncertainties in the inner $| y |_{\text {max}} < 1$ (*solid line*) and in the outer rapidity region $1 \le | y |_{\text {max}} < 2$ (*dashed line*)

For the NP corrections, the multijet-improved MC event generators sherpa version 1.4.3 [45] and MadGraph  5 version 1.5.12 [46] are used to simulate 3-jet events. sherpa employs a dipole formulation for parton showering [47, 48], a cluster model for hadronization [49], and an MPI model for the underlying event that is based on independent hard processes similar to pythia  [33, 45]. In the case of MadGraph, the steps of parton showering, hadronization, and multiple parton scatterings come from pythia version 6.4.26 with default settings using the Lund string model for hadronization [29–31] and a multiple-interaction model for the underlying event that is interleaved with the parton shower [27]. The 3-jet mass is determined for a given event before and after the MPI and hadronization phases are performed. This allows the derivation of correction factors, which are applied to the theory prediction at NLO. The correction factor is defined as the mean of the corrections from the two examined event generators and ranges in value from 1.16 for the low mass range to about 1.05 at high 3-jet mass. The systematic uncertainty in the NP correction factors is estimated as plus or minus half of the spread between the two predictions and amounts to roughly $\pm$2 %. The NP correction factors and their uncertainties are shown in Fig. 2 for both rapidity bins.

**Fig. 3:**
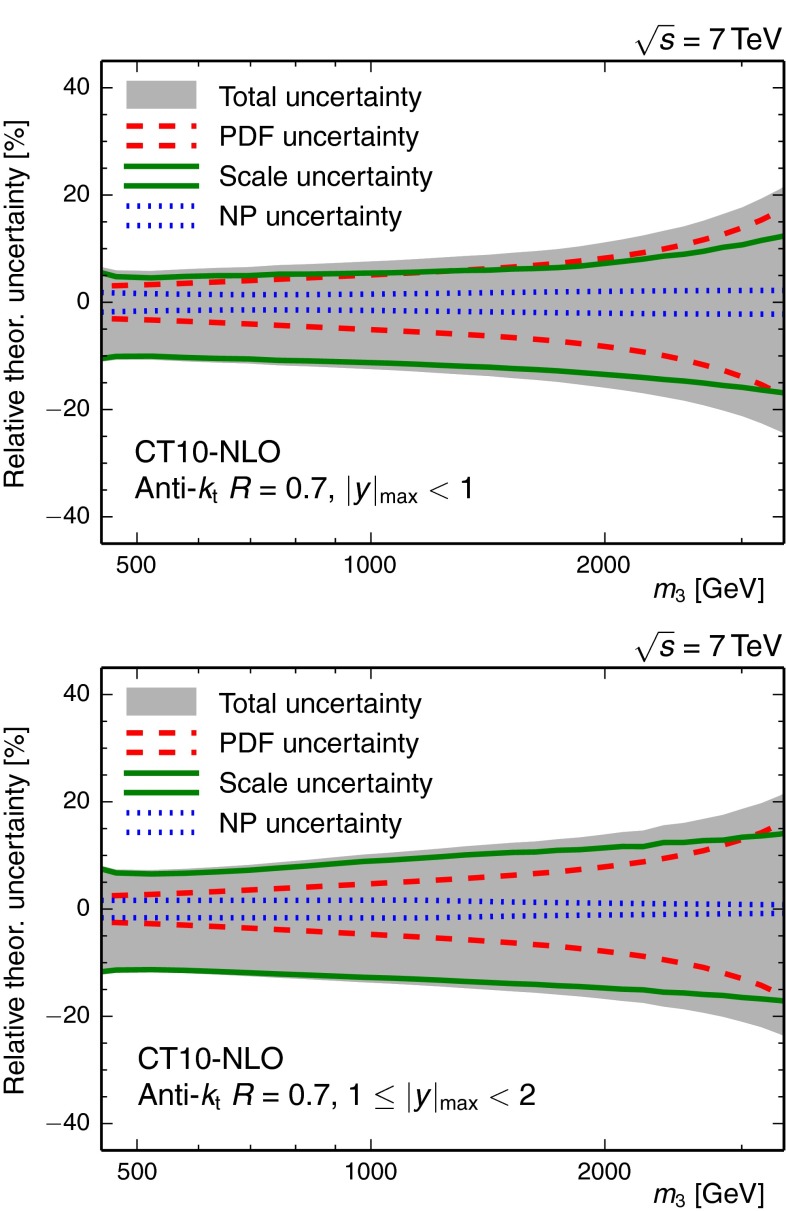
Overview of the theory uncertainties in the inner $| y |_{\text {max}} < 1$ (*top*) and in the outer rapidity region $1 \le | y |_{\text {max}} < 2$ (*bottom*) for the CT10 PDF set with NLO PDF evolution

An overview of the different theoretical uncertainties is given in Fig. 3.

## Results and determination of the strong coupling constant

**Fig. 4:**
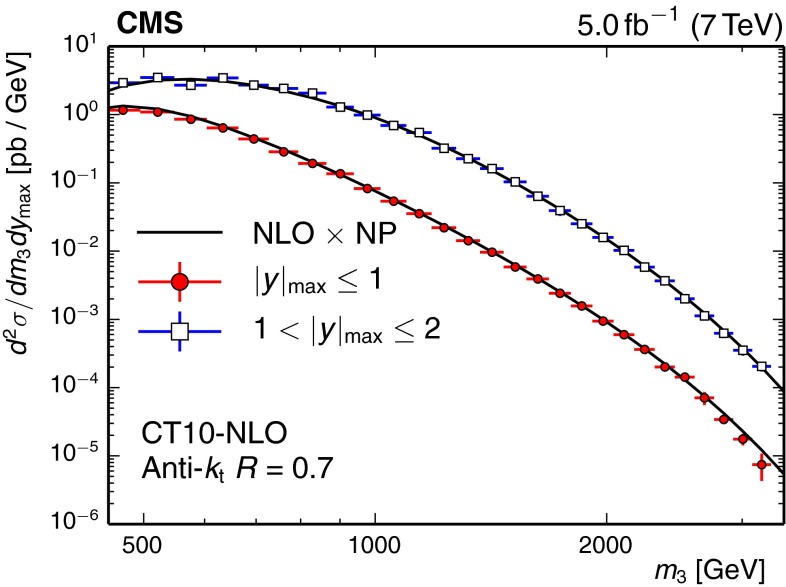
Comparison of the measured 3-jet mass cross section with the theory prediction for the two regions in $| y |_{\text {max}}$. This prediction is based on an NLO 3-jet calculation, which employs the CT10-NLO PDF set and is corrected for nonperturbative effects. The *vertical error bars* represent the total experimental uncertainty, while the *horizontal error bars* indicate the bin widths

**Fig. 5:**
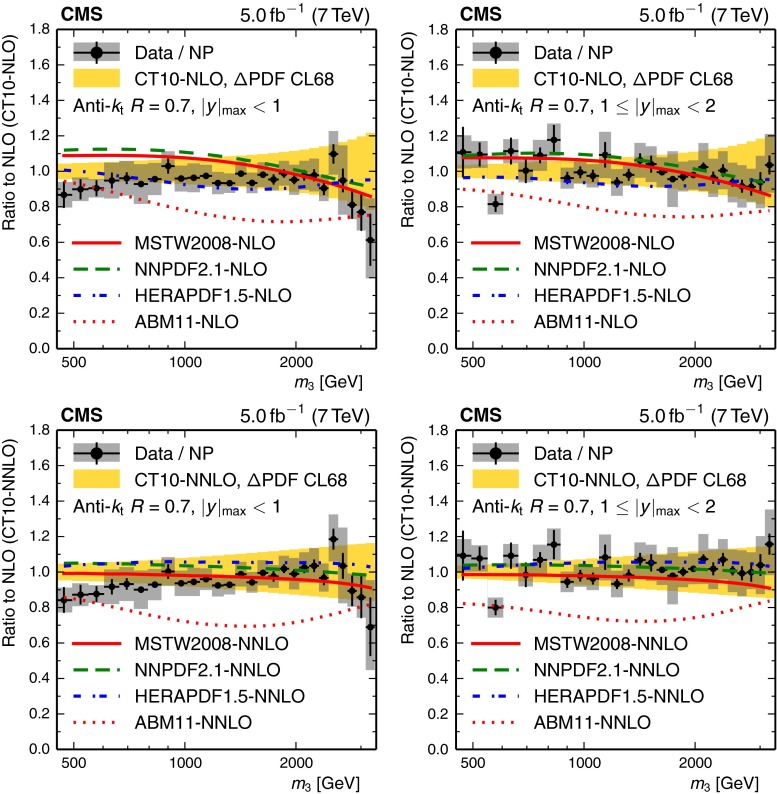
Ratio of the 3-jet mass cross section, divided by NP corrections, to the theory prediction at NLO with the CT10-NLO (*top*) or CT10-NNLO PDF set (*bottom*) for the inner rapidity region (*left*) and for the outer rapidity region (*right*). The data are shown with *error bars* representing the statistical uncertainty after unfolding added quadratically to the 1 % uncorrelated residual uncertainty and *gray rectangles* for the total correlated systematic uncertainty. The *light gray* (colour version: *yellow*) *band* indicates the PDF uncertainty for the CT10 PDF sets at 68 % confidence level. In addition, the ratios of the NLO predictions are displayed for the PDF sets MSTW2008, NNPDF2.1, HERAPDF1.5, and ABM11, also at next-to- (*top*) and next-to-next-to-leading evolution order (*bottom*)

Figure 4 compares the measured 3-jet mass spectrum to the Theory prediction. This prediction is based on an NLO 3-jet calculation, which employs the CT10-NLO PDF set and is corrected for nonperturbative effects. Perturbative QCD describes the 3-jet mass cross section over five orders of magnitude for 3-jet masses up to 3 TeV. The ratios of the measured cross sections to the theory predictions are presented in Fig. 5 to better judge potential differences between data and theory. Within uncertainties, most PDF sets are able to describe the data. Some deviations are visible for small $m_3$. Significant deviations are exhibited when using the ABM11 PDFs, which therefore are not considered in our fits of $\alpha _S(M_\mathrm{Z})$.

**Fig. 6:**
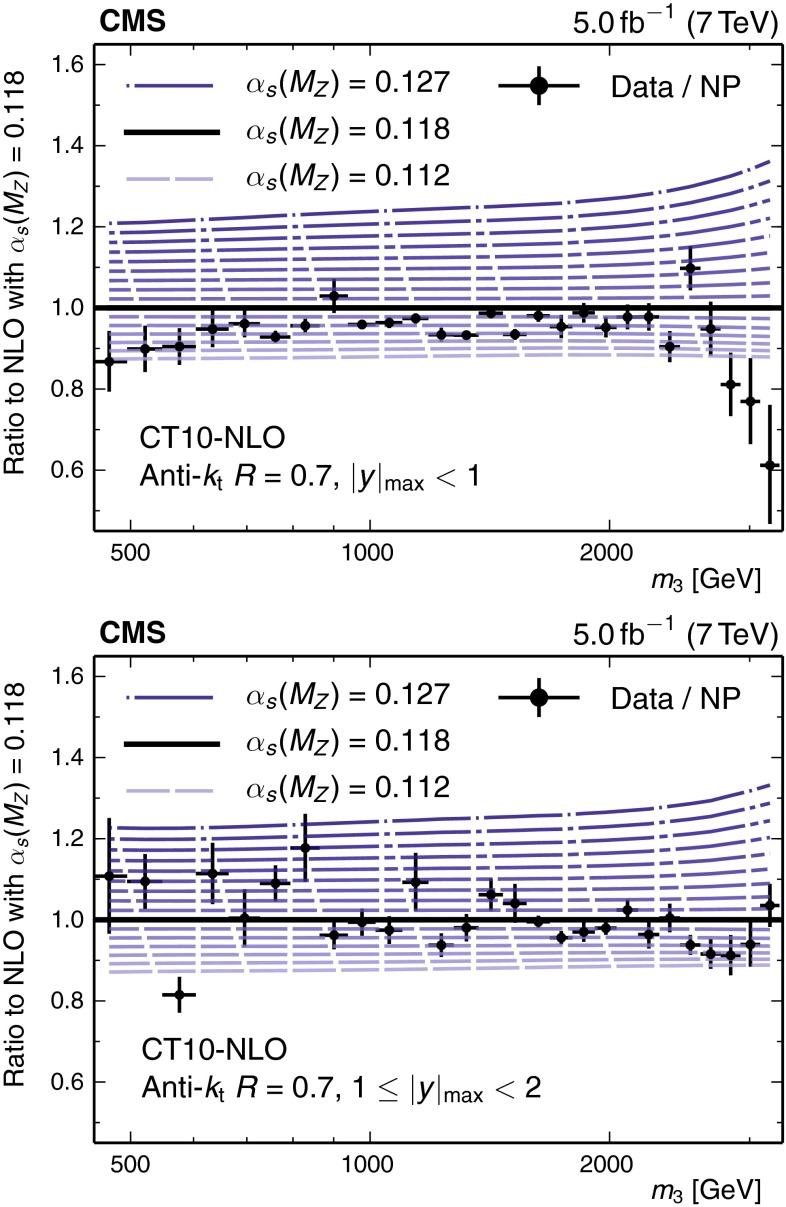
Ratio of the measured 3-jet mass cross section in the inner rapidity region (*top*) and in the outer rapidity region (*bottom*), divided by the NP correction, with respect to the theory prediction at NLO while using the CT10-NLO PDF set with the default value of $\alpha _S(M_\mathrm{Z}) = 0.118$. In addition, ratios are shown for the theory predictions with CT10-NLO PDFs assuming values of $\alpha _S(M_\mathrm{Z})$ ranging from 0.112 up to 0.127 in steps of 0.001. The *error bars* represent the total uncorrelated uncertainty of the data

In the following, the PDFs are considered to be an external input such that a value of $\alpha _S(M_\mathrm{Z})$ can be determined. Potential correlations between $\alpha _S(M_\mathrm{Z})$ and the PDFs are taken into account by using PDF sets that include variations in $\alpha _S(M_\mathrm{Z})$ as listed in Table 2. Figure 6 demonstrates for the example of the CT10-NLO PDF set the sensitivity of the theory predictions with respect to variations in the value of $\alpha _S(M_\mathrm{Z})$ in comparison to the data and their total uncertainty.

A value of $\alpha _S(M_\mathrm{Z})$ is determined by minimizing the $\chi ^2$ between the $N$ measurements $D_i$ and the theoretical predictions $T_i$. The $\chi ^2$ is defined as

4 $$\begin{aligned} \chi ^2 = \sum _{ij}^N \left( D_i - T_i\right) \mathrm {C}_{ij}^{-1} \left( D_j - T_j\right) , \end{aligned}$$

where the covariance matrix $C_{ij}$ is composed of the following terms:

5 $$\begin{aligned} C= & {} {{\mathrm{cov}}}_{\text {unf}+\text {stat}} + {{\mathrm{cov}}}_{\text {uncor}} \nonumber \\&+\left( \sum _\text {sources}{{\mathrm{cov}}}_\mathrm {JES}\right) + {{\mathrm{cov}}}_{\text {lumi}} + {{\mathrm{cov}}}_{\mathrm {PDF}}, \end{aligned}$$

6 $$\begin{aligned} C = {{\mathrm{cov}}}_{\text {unf}+\text {stat}} + {{\mathrm{cov}}}_{\text {uncor}} + \left( \sum _\text {sources}{{\mathrm{cov}}}_\mathrm {JES}\right) + {{\mathrm{cov}}}_{\text {lumi}} + {{\mathrm{cov}}}_{\mathrm {PDF}}, \end{aligned}$$

and the terms in the sum represent

1. ${{\mathrm{cov}}}_{\text {unf}+\text {stat}}$: statistical and unfolding uncertainty including correlations induced through the unfolding;
2. ${{\mathrm{cov}}}_\text {uncor}$: uncorrelated systematic uncertainty summing up small residual effects such as trigger and identification inefficiencies, time dependence of the jet $p_{\mathrm {T}}$ resolution, and the uncertainty on the trigger prescale factor;
3. ${{\mathrm{cov}}}_{\mathrm {JES},\text {sources}}$: systematic uncertainty for each JES uncertainty source;
4. ${{\mathrm{cov}}}_\text {lumi}$: luminosity uncertainty; and
5. $${{\mathrm{cov}}}_\mathrm {PDF}$$: PDF uncertainties.

The first four sources constitute the experimental uncertainty. The JES and luminosity uncertainty are treated as fully correlated across the $m_3$ and $| y |_{\text {max}}$ bins, where for the JES uncertainty the procedure recommended in Ref. [12] is applied. The derivation of PDF uncertainties follows prescriptions for each individual PDF set. The CT10 and MSTW PDF sets both employ the Hessian or eigenvector method [50] with upward and downward variations for each eigenvector. As required by the use of covariance matrices, symmetric PDF uncertainties are computed following Ref. [51]. For the HERAPDF1.5 PDF set, which employs a Hessian method for the experimental uncertainties, complemented with model and parameterization uncertainties, the prescription from Ref. [41] is used. The NNPDF2.1 PDF set uses the technique of MC pseudo-experiments instead of the eigenvector method to provide PDF uncertainties. The ensemble of replicas, whose averaged predictions give the central result, are evaluated following the prescription in Ref. [52] to derive the PDF uncertainty for NNPDF. The JES and luminosity uncertainties are assumed to be multiplicative to avoid the statistical bias that arises from uncertainty estimations taken from data [53–55]. The uncertainty in a result for $\alpha _S(M_\mathrm{Z})$ from a $\chi ^2$ fit is obtained from the $\alpha _S(M_\mathrm{Z})$ values for which the $\chi ^2$ is increased by one with respect to the minimum value.

The uncertainty in $\alpha _S(M_\mathrm{Z})$ due to the NP uncertainties is evaluated by looking for maximal offsets from a default fit. The theoretical prediction $T$ is varied by the NP uncertainty $\Delta \mathrm {NP}$ as $T\cdot \mathrm {NP} \rightarrow T\cdot \left( \mathrm {NP} \pm \Delta \mathrm {NP}\right)$. The fitting procedure is repeated for these two variations, and the deviation from the central $\alpha _S(M_\mathrm{Z})$ values is considered as the uncertainty in $\alpha _S(M_\mathrm{Z})$. Finally, the uncertainty due to the $\mu _r$ and $\mu _f$ scales is evaluated by applying the same method as for the NP corrections, varying $\mu _r$ and $\mu _f$ by the six scale factor combinations as described in Sect. 5.

The shape of the predicted 3-jet mass cross section depends on the QCD matrix elements and kinematic constraints. Because each of the leading three jets is required to have a $p_{\mathrm {T}}$ larger than 100 GeV, some event configurations, possible with respect to the QCD matrix elements, are kinematically forbidden at low $m_3$. In the spectra shown in Fig. 4, this fact is visible in the form of a maximum in the 3-jet mass cross section, which is shifted to higher $m_3$ values for the outer compared to the inner $| y |_{\text {max}}$ bin because the larger differences in the jet rapidities allow higher $m_3$ to be reached with lower $p_{\mathrm {T}}$ jets. For fits of $\alpha _S(M_\mathrm{Z})$ the $m_3$ region limited through kinematical constraints is unsuited, since close to the phase space boundaries fixed-order pQCD calculations might be insufficient and resummations might be required. To avoid this region of phase space as done in Ref. [11], only $m_3$ bins beyond the maximum of the 3-jet mass cross section in the outer $| y |_{\text {max}}$ bin are considered. This corresponds to a minimum in $m_3$ of 664 GeV. Including one bin more or less induces changes in the measured $\alpha _S(M_\mathrm{Z})$ below the percent level. To study the running of the strong coupling, the comparison between data and theory is also performed in several 3-jet mass regions above 664 GeV as shown in Table 3.

For the evolution of $\alpha _\mathrm {S}(Q)$ in the fits of $\alpha _S(M_\mathrm{Z})$, the Glück–Reya–Vogt formula [56] is used at 2-loop order as implemented in fastNLO. The capability of fastNLO to replace the $\alpha _\mathrm {S}(Q)$ evolution of a PDF set by such alternative codes is exploited to interpolate cross section predictions between the available fixed points of $\alpha _S(M_\mathrm{Z})$ listed in Table 2. Limited extrapolations beyond the lowest or highest values of $\alpha _S(M_\mathrm{Z})$ provided in a PDF series are accepted if necessary for uncertainty evaluations, up to a limit of $|\Delta \alpha _S(M_\mathrm{Z}) | = 0.003$. This extrapolation method can be necessary in some cases to fully evaluate the scale uncertainty. The procedure has been cross-checked using the original $\alpha _\mathrm {S}(Q)$ grid of each PDF within LHAPDF and with the evolution code of the hoppet toolkit [57] and of RunDec  [58, 59].

The CT10-NLO PDF set is chosen for the main result for two reasons: The range in available $\alpha _S(M_\mathrm{Z})$ values is wide enough to evaluate almost all scale uncertainties within this range and the central value of $\alpha _S(M_\mathrm{Z})$ in this set is rather close to the combined fit result.

The fit results for $\alpha _S(M_\mathrm{Z})$ and $\alpha _\mathrm {S}(Q)$ for all considered $m_3$ ranges are presented in Tables 3 and 4, respectively. Fits over the total $m_3$ range above 664 GeV are shown for each $y_{\text {max}}$ bin separately and for both combined in the bottom three rows of Table 3.

**Table 3:** Determinations of $\alpha _S(M_\mathrm{Z})$ in the considered $m_3$ ranges. The relevant scale in each 3-jet mass range is calculated from the cross section-weighted average as given by the theory prediction using the CT10 PDF set with NLO evolution. The three bottom rows present fits using the whole 3-jet mass range above 664 GeV in both rapidity regions either separately or combined (last row). Uncertainties are quoted separately for experimental sources, the PDFs, the NP corrections, and the scale uncertainty

$m_3$ ($\text {GeV}$)	$\left<Q\right>$ ($\text {GeV}$)	$\chi ^2/n_\mathrm {dof}$	$\alpha _S(M_\mathrm{Z})$	$\pm \text {(exp)}$	$\pm \mathrm {(PDF)}$	$\pm \mathrm {(NP)}$	$\pm \text {(scale)}$
$$ 664$$–$$ 794$$	$$ 361 $$	$$ 4.5 / 3$$	$$0.1232$$	$$^{+0.0040}_{-0.0042} $$	$$^{+0.0019}_{-0.0016} $$	$$^{+0.0008}_{-0.0007} $$	$$^{+0.0079}_{-0.0044} $$
$$ 794$$–$$ 938$$	$$ 429 $$	$$ 7.8 / 3$$	$$0.1143$$	$$^{+0.0034}_{-0.0033} $$	$$^{+0.0019}_{-0.0016} $$	$$\pm 0.0008 $$	$$^{+0.0073}_{-0.0042} $$
$$ 938$$–$$1098$$	$$ 504 $$	$$ 0.6 / 3$$	$$0.1171$$	$$^{+0.0033}_{-0.0034} $$	$$\pm 0.0022 $$	$$\pm 0.0007 $$	$$^{+0.0068}_{-0.0040} $$
$$1098$$–$$1369$$	$$ 602 $$	$$ 2.6 / 5$$	$$0.1152$$	$$\pm 0.0026 $$	$$^{+0.0027}_{-0.0026} $$	$$^{+0.0008}_{-0.0007} $$	$$^{+0.0060}_{-0.0027} $$
$$1369$$–$$2172$$	$$ 785 $$	$$ 8.8 / 13$$	$$0.1168$$	$$^{+0.0018}_{-0.0019} $$	$$^{+0.0030}_{-0.0031} $$	$$^{+0.0007}_{-0.0006} $$	$$^{+0.0068}_{-0.0034} $$
$$2172$$–$$2602$$	$$1164 $$	$$ 3.6 / 5$$	$$0.1167$$	$$^{+0.0037}_{-0.0044} $$	$$^{+0.0040}_{-0.0044} $$	$$\pm 0.0008 $$	$$^{+0.0065}_{-0.0041} $$
$$2602$$–$$3270$$	$$1402 $$	$$ 5.5 / 7$$	$$0.1120$$	$$^{+0.0043}_{-0.0041} $$	$$^{+0.0056}_{-0.0040} $$	$$\pm 0.0001 $$	$$^{+0.0088}_{-0.0050} $$
$$| y |_{\text {max}} < 1$$	$$ 413 $$	$$10.3 / 22$$	$$0.1163$$	$$^{+0.0018}_{-0.0019} $$	$$\pm 0.0027 $$	$$\pm 0.0007 $$	$$^{+0.0059}_{-0.0025} $$
$$1\le | y |_{\text {max}} <2$$	$$ 441 $$	$$10.6 / 22$$	$$0.1179$$	$$^{+0.0018}_{-0.0019} $$	$$\pm 0.0021 $$	$$\pm 0.0007 $$	$$^{+0.0067}_{-0.0037} $$
$$| y |_{\text {max}} < 2$$	$$ 438 $$	$$47.2 / 45$$	$$0.1171$$	$$\pm 0.0013 $$	$$\pm 0.0024 $$	$$\pm 0.0008 $$	$$^{+0.0069}_{-0.0040} $$

**Table 4:** Same as Table 3 but showing the fit result in terms of $\alpha _\mathrm {S}(Q)$ for each range in $Q$

$m_3$ ($\text {GeV}$ )	$\left<Q\right>$ ($\text {GeV}$ )	$\chi ^2/n_\mathrm {dof}$	$\alpha _\mathrm {S}(Q)$	$\pm \text {(exp)}$	$\pm \mathrm {(PDF)}$	$\pm \mathrm {(NP)}$	$\pm \text {(scale)}$
$$ 664$$–$$ 794$$	$$ 361 $$	$$ 4.5 / 3$$	$$0.1013$$	$$^{+0.0027}_{-0.0028} $$	$$^{+0.0013}_{-0.0011} $$	$$\pm 0.0005 $$	$$^{+0.0052}_{-0.0030} $$
$$ 794$$–$$ 938$$	$$ 429 $$	$$ 7.8 / 3$$	$$0.0933$$	$$\pm 0.0022 $$	$$^{+0.0012}_{-0.0011} $$	$$\pm 0.0005 $$	$$^{+0.0048}_{-0.0028} $$
$$ 938$$–$$1098$$	$$ 504 $$	$$ 0.6 / 3$$	$$0.0934$$	$$\pm 0.0021 $$	$$\pm 0.0014 $$	$$\pm 0.0005 $$	$$^{+0.0043}_{-0.0025} $$
$$1098$$–$$1369$$	$$ 602 $$	$$ 2.6 / 5$$	$$0.0902$$	$$\pm 0.0016 $$	$$\pm 0.0016 $$	$$^{+0.0005}_{-0.0004} $$	$$^{+0.0036}_{-0.0017} $$
$$1369$$–$$2172$$	$$ 785 $$	$$ 8.8 / 13$$	$$0.0885$$	$$^{+0.0010}_{-0.0011} $$	$$^{+0.0017}_{-0.0018} $$	$$^{+0.0004}_{-0.0003} $$	$$^{+0.0038}_{-0.0020} $$
$$2172$$–$$2602$$	$$1164 $$	$$ 3.6 / 5$$	$$0.0848$$	$$^{+0.0019}_{-0.0023} $$	$$^{+0.0020}_{-0.0023} $$	$$\pm 0.0004 $$	$$^{+0.0034}_{-0.0021} $$
$$2602$$–$$3270$$	$$1402 $$	$$ 5.5 / 7$$	$$0.0807$$	$$^{+0.0022}_{-0.0021} $$	$$^{+0.0028}_{-0.0021} $$	$$\pm 0.0001 $$	$$^{+0.0044}_{-0.0026} $$

For comparison, the combined fit was also tried for alternative PDF sets listed in Table 5. For the ABM11 PDFs, which predict 3-jet mass cross sections that are too small, fits are technically possible. However, to compensate for this discrepancy, the $\alpha _S(M_\mathrm{Z})$ results take unreasonably high values that are far outside the $\alpha _S(M_\mathrm{Z})$ values that are given by the PDF authors. For the NNPDF2.1-NLO and HERAPDF1.5-NLO PDF series, a central value for $\alpha _S(M_\mathrm{Z})$ can be calculated, but the range in $\alpha _S(M_\mathrm{Z})$ values is not sufficient for a reliable determination of uncertainty estimations. In all other cases the fit results for $\alpha _S(M_\mathrm{Z})$ are in agreement between the investigated PDF sets and PDF evolution orders within uncertainties.

**Table 5:** Determinations of $\alpha _S(M_\mathrm{Z})$ with different PDF sets using all 3-jet mass points with $m_3 > 664\hbox { GeV}$. Uncertainties are quoted separately for experimental sources, the PDFs, the NP corrections, and the scale uncertainty

PDF set	$\chi ^2/n_\mathrm {dof}$	$\alpha _S(M_\mathrm{Z})$	$\pm \text {(exp)}$	$\pm \mathrm {(PDF)}$	$\pm \mathrm {(NP)}$	$\pm \,\text {(scale)}$
CT10-NLO	$$47.2 / 45$$	$$0.1171$$	$$\pm 0.0013 $$	$$\pm 0.0024 $$	$$\pm 0.0008 $$	$$^{+0.0069}_{-0.0040} $$
CT10-NNLO	$$48.5 / 45$$	$$0.1165$$	$$^{+0.0011}_{-0.0010} $$	$$^{+0.0022}_{-0.0023} $$	$$^{+0.0006}_{-0.0008} $$	$$^{+0.0066}_{-0.0034} $$
MSTW2008-NLO	$$52.8 / 45$$	$$0.1155$$	$$^{+0.0014}_{-0.0013} $$	$$^{+0.0014}_{-0.0015} $$	$$^{+0.0008}_{-0.0009} $$	$$^{+0.0105}_{-0.0029} $$
MSTW2008-NNLO	$$53.9 / 45$$	$$0.1183$$	$$^{+0.0011}_{-0.0016} $$	$$^{+0.0012}_{-0.0023} $$	$$^{+0.0011}_{-0.0019} $$	$$^{+0.0052}_{-0.0050} $$
HERAPDF1.5-NNLO	$$49.9 / 45$$	$$0.1143$$	$$\pm 0.0007 $$	$$^{+0.0020}_{-0.0035} $$	$$^{+0.0003}_{-0.0008} $$	$$^{+0.0035}_{-0.0027} $$
NNPDF2.1-NNLO	$$51.1 / 45$$	$$0.1164$$	$$\pm 0.0010 $$	$$^{+0.0020}_{-0.0019} $$	$$^{+0.0010}_{-0.0009} $$	$$^{+0.0058}_{-0.0025} $$

**Fig. 7:**
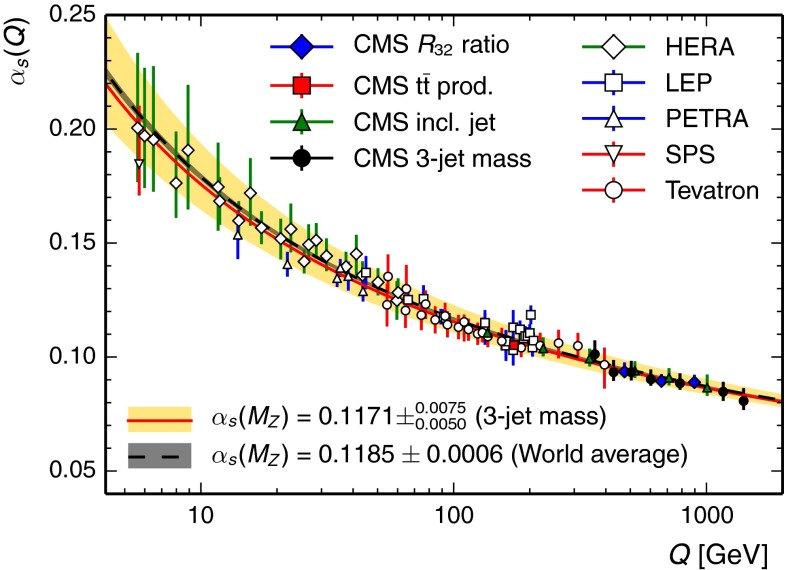
Comparison of the $\alpha _\mathrm {S}(Q)$ evolution as determined in this analysis from all measurement bins with $m_3 > 664\hbox { GeV}$ (*solid curve* with *light grey* uncertainty band; colour version: *red curve* with *yellow* uncertainty band) to the world average (*dashed curve* with *dark grey* uncertainty band) [60]. The *error bars* on the data points correspond to the total uncertainty. In addition, an overview of measurements of the running of the strong coupling $\alpha _\mathrm {S}(Q)$ from electron–positron [65–67], electron–proton [69–72], and proton–(anti)proton collider experiments [11, 61, 62, 68] is presented. The results of this analysis extend the covered range in values of the scale $Q$ up to ${\approx }$1.4 TeV

Figure 7 shows the $\alpha _\mathrm {S}(Q)$ evolution determined in this analysis with CT10-NLO in comparison to the world average of $\alpha _S(M_\mathrm{Z}) = 0.1185 \pm 0.0006$ [60]. The figure also shows an overview of the measurements of the running of the strong coupling from various other experiments [61–67] together with recent determinations by CMS [11, 12, 68] and from this analysis. Within uncertainties, the new results presented here are in agreement with previous determinations and extend the covered range in scale $Q$ up to a value of 1.4 TeV.

## Summary

The proton–proton collision data collected by the CMS experiment in 2011 at a centre-of-mass energy of 7 TeV were used to measure the double-differential 3-jet production cross section as a function of the invariant mass $m_3$ of the three jets leading in $p_{\mathrm {T}}$, and of their maximum rapidity $y_{\text {max}}$. The measurement covers a 3-jet mass range from 445 GeV up to 3270 GeV in two bins of rapidity up to $|y_{\text {max}} | = 2$. Within experimental and theoretical uncertainties, which are of comparable size, the data are in agreement with predictions of perturbative QCD at next-to-leading order.

The strong coupling constant has been determined in multiple regions of 3-jet mass for values of the scale $Q$ between 0.4 and 1.4 TeV from a comparison between data and theory. The results are consistent with the evolution of the strong coupling as predicted by the renormalization group equation and extend the range in $Q$ where this could be tested up to 1.4 TeV. A combined fit of all data points above a 3-jet mass of 664 GeV gives the value of the strong coupling constant $\alpha _S(M_\mathrm{Z}) = 0.1171 \pm 0.0013\,\text {(exp)} \pm 0.0024\,(\mathrm {PDF}) \pm 0.0008\,(\mathrm {NP}) \,^{+0.0069}_{-0.0040}\,\text {(scale)}$.

This result, achieved with 3-jet production cross sections, is consistent with determinations previously reported by CMS using the inclusive jet cross section [12] and the ratio of inclusive 3-jet to inclusive 2-jet production cross sections [11]. It is also consistent with a recent determination of $\alpha _S(M_\mathrm{Z})$ by CMS at the top production threshold using theory at NNLO [68] and with the latest world average of $\alpha _S(M_\mathrm{Z}) = 0.1185 \pm 0.0006$ [60].
